# Intra-arrest strategies to treat cardiac arrest due to a non-opioid toxicological causes in adults and children: a systematic review

**DOI:** 10.1016/j.resplu.2026.101501

**Published:** 2026-09-24

**Authors:** Keith Couper, Amani Alenazi, James Gray, Brian Grunau, Stephan Katzenschlager, Ben Rutherford, Michael D. Simpson, Ian R. Drennan, Barney Scholefield, Eric J. Lavonas, Advanced Life Support and Pediatric Life Support Task Forces of the International Liaison Committee on Resuscitation, Anders Aneman, Bernd W. Böttiger, Yew Woon Chia, Conor Crowley, Sonia D’Arrigo, Charles Deakin, Shannon M. Fernando, Asger Granfeldt, Karen Hirsch, Mathias J. Holmberg, Peter J. Kudenchuk, Carrie Leong, Ari Moskowitz, Robert W. Neumar, Tonia Nicholson, Nikolaos Nikoloau, Jerry P. Nolan, Shinichiro Ohshimo, Brian J. O’Neil, Michael Parr, Sarah M. Perman, Helen Pocock, Claudio Sandroni, Tommaso Scquizzato, Markus B. Skrifvars, Jasmeet Soar, Neville Vlok, Michelle Welsford, Johannes Wittig, Carolyn M. Zelop, Jason Acworth, Lokesh Kumar Tiwari, Tia T. Raymond, Andrea Christoff, Arun Bansal, Alexis Topjian, Monica Kleinman, Hiroshi Kurosawa, Michelle Myburgh, Jimena del Castillo, Joseph Rossano, Ryan W. Morgan, Leandra Rech, Anne-Marie Guerguerian, Vinay Nadkarni, Thomaz Bittencourt Couto, Stephen M. Schexnayder, Gabrielle Nuthall, Janice A. Tijssen, Gene Yong-Kwang Ong, Ester Ambunda, Laurie J. Morrison, Dianne L. Atkins, Allan R. de Caen

**Affiliations:** a Warwick Medical School, University of Warwick, Coventry, UK; b Emergency Medical Services Department, College of Applied Medical Sciences, King Saud Bin Abdulaziz University for Health Sciences, Riyadh, Saudi Arabia; c King Abdullah International Medical Research Centre, Riyadh, Saudi Arabia; d Division of Emergency Medicine, Cincinnati Children’s Hospital Medical Center, Cincinnati, OH, USA; e Department of Pediatrics, University of Cincinnati, Cincinnati, OH, USA; f Centre for Advancing Health Outcomes, St. Paul’s Hospital, Vancouver, British Columbia, Canada; g Department of Emergency Medicine, University of British Columbia, Vancouver, British Columbia, Canada; h British Columbia Emergency Health Services, Vancouver, British Columbia, Canada; i Heidelberg University, Medical Faculty Heidelberg, Department of Anaesthesiology, Heidelberg, Germany; j Department of Emergency Medicine, Beth Israel Deaconess Medical Center, Boston, MA, USA; k Massachusetts and Rhode Island Poison Center, Boston, MA, USA; l Interdepartmental Division of Critical Care Medicine, Department of Medicine, University of Toronto, Toronto, Canada; m Department of Emergency Services and Sunnybrook Research Institute, Sunnybrook Health Science Centre, Canada; n Ornge Air Ambulance and Critical Care Transport, Toronto, Canada; o Department of Critical Care Medicine, The Hospital for Sick Children, Toronto, Canada; p Rocky Mountain Poison and Drug Safety, Denver Health, Denver, CO, USA; q Department of Emergency Medicine, University of Colorado School of Medicine, Aurora, CO, USA

**Keywords:** Cardiac arrest, Cardiopulmonary resuscitation, Toxicology, Poisons, Systematic review

## Abstract

**Objective:**

To summarise evidence on the effectiveness of specific alterations to standard advanced life support during ongoing cardiopulmonary resuscitation in adults and children with cardiac arrest due to a presumed toxicological cause.

**Methods:**

We searched MEDLINE (OVID platform), Embase (OVID platform), and Cochrane CENTRAL from inception to 22nd July 2026 for relevant observational and interventional studies with a control group. We excluded studies focussed specifically on opioid toxicity. Two authors independently screened studies for inclusion. Our primary outcome was 30-day survival. We assessed risk of bias using the Cochrane recommended tools and certainty of evidence using the GRADE system.

**Results:**

We identified four observational studies, of which none specifically focused on children and three included fewer than 100 patients. Studies examined general toxicological causes (*n* = 2), smoke inhalation and cyanide poisoning (*n* = 1), and beta-blocker and calcium-channel blocker overdose (*n* = 1). All studies were at critical risk of bias.

Across the four studies, nine interventions were reported. Whilst some interventions were associated with either benefit or harm, estimates of effect were very imprecise and challenging to interpret. The largest study (1545 patients) reported that sodium bicarbonate use in out-of-hospital cardiac arrest due to suspected drug overdose was associated with lower survival to hospital discharge (adjusted odds ratio 0.16, 95% confidence interval 0.08–0.31).

**Conclusion:**

Current evidence does not support the modification of standard advanced life support during cardiac arrest due to a presumed toxicological cause in adults or children.

**Registration:** PROSPERO CRD420251149412.

## Introduction

Cardiac arrest due to toxicological causes is infrequent, with incidence across studies ranging from 0.26 to 4.25 events per 100,000 person-years and varying across settings.^1^ The proportion of cardiac arrests attributed to a toxicological cause ranges from around 1% for paediatric in-hospital and out-of-hospital cardiac arrest to 8% for adult out-of-hospital cardiac arrest.^2^, ^3^, ^4^ Compared with other causes of out-of-hospital cardiac arrest, cases attributable to overdose more often present in a non-shockable rhythm and are unwitnessed.^3^, ^5^ Despite this, outcomes in this patient cohort are often better than the general cardiac arrest population, potentially reflecting more favourable patient characteristics, such as younger age, and the potential reversibility of the cardiac arrest cause.^3^, ^5^ In addition, some patients who have an organized electrical rhythm but no palpable pulses may be hypotensive but not in cardiac arrest (“pseudo-PEA”).^6^, ^7^

Across international adult and paediatric resuscitation guidelines, toxins are identified as a potentially “reversible cause” of cardiac arrest.^8^, ^9^, ^10^, ^11^ A key challenge for clinicians is knowing if there are actually treatments that can “reverse” a toxin-related cardiac arrest, and when there is a need to modify standard Advanced Life Support to treat the presumed toxicological cause. In the context of cardiac arrest, key considerations may include uncertainty as to a specific toxicological cause, the immediate availability of any antidote, and uncertainty as to the effectiveness of that antidote in cardiac arrest. For example, in the context of opioid-induced cardiac arrest, the administration of opioid antagonists is not consistently associated with improved clinical outcome.^12^ This may reflect the fact that the primary mechanism through which opioids cause cardiac arrest is through respiratory depression and associated hypoxaemia, which is treated in cardiac arrest through ventilation and supplemental oxygen.^13^

Historically, the International Liaison Committee on Resuscitation (ILCOR) has reviewed the evidence for specific toxins in isolation, including beta-blockers, calcium channel blockers, and local anaesthetics.^14^, ^15^ In addition to cardiac arrest management, previous ILCOR reviews have also included management of life-threatening toxin-induced respiratory depression or hypotension. A more recent ILCOR systematic review focused specifically on opioid-induced cardiac arrest.^12^ We undertook this systematic review to address the knowledge gap that exists for intra-arrest strategies to treat cardiac arrest for all other toxicological causes.

## Methods

This systematic review was prospectively registered with PROSPERO (CRD420251149412), undertaken in line with ILCOR methodological guidelines, and is reported in line with the Preferred Reporting Items for Systematic reviews and Meta-Analyses (PRISMA) reporting guideline.^16^ The ILCOR review template is included in the supplementary materials. A formal review protocol was not prepared. The review is consistent with the PROSPERO registration. All available data and materials are included within the review manuscript.

Our overarching research question was:

In adults and children in any setting (in-hospital or out-of-hospital) with cardiac arrest due to a presumed toxicological cause, does any specific alteration to standard Advanced Life Support during ongoing cardiopulmonary resuscitation compared with either standard Advanced Life Support or any other specific alteration to standard Advanced Life Support during ongoing cardiopulmonary resuscitation improve clinical outcomes?

### Study eligibility criteria

We included studies that evaluated the clinical effect of any modification to standard advanced life support during ongoing cardiopulmonary resuscitation of an adult or child in cardiac arrest due to a presumed toxicological cause other than opioids.

Eligible studies were randomised or non-randomised studies with a relevant control group, such as randomised controlled trials, non-randomised controlled trials, interrupted time series, controlled before-and-after studies, cohort studies. We excluded studies without a control group, unpublished studies (e.g., conference abstracts, trial protocols), studies where an abstract was not available in English, and non-human studies (e.g. animal, simulation studies).

We identified specific toxins of interest based on previous ILCOR reviews and task force member expertise on important toxins in their clinical setting, including: beta-blockers, carbon monoxide, digoxin/cardiac glycosides, jellyfish envenomation, and tricyclic antidepressants. The full list of toxins of interest is available in the supplementary materials. Studies looking specifically at opioids were excluded as interventions for opioid-induced cardiac arrest were evaluated in a recent ILCOR systematic review.^12^

### Searches

We initially searched MEDLINE (OVID platform), EMBASE (OVID platform), and the Cochrane Library on December 2025, and updated our search on 22nd July 2026 (see Supplementary Materials). Our search strategy, which was originally developed in MEDLINE and subsequently translated into syntax for other databases, incorporated index and free-terms terms for cardiac arrest, specific toxins of interest, and poisoning. Parts of our search strategy were based on the searches developed for recent American Heart Association (AHA) cardiac arrest guidelines.^17^, ^18^ We sought to identify additional relevant studies through searching clinical trial registers and forward and backward citation tracking of included studies.

### Outcomes

Our primary outcome was survival at 30-days. In the event that survival at 30-days was not reported in a specific study, we planned to include studies that reported survival at hospital discharge on the basis that previous studies suggest these outcomes are comparable.^19^

Secondary outcomes included: health-related quality of life (measured at 3-months, 6-months or longest follow-up), survival with favourable neurological outcome (measured at hospital discharge/30-days, 3-months, 6-months or longest follow-up), survival (measured at hospital discharge, 3-months, 6-months or longest follow-up), sustained return of spontaneous circulation (hospital handover or 20-min), and any return of spontaneous circulation (ROSC).

In line with GRADE methodology and the core outcome set for cardiac arrest in adults and children, we categorised survival at 30-days, survival with favourable neurological outcome at 30-days/discharge, and health-related quality of life at 3-months are designated as critical outcomes.^20^, ^21^, ^22^ All other outcomes are designated as important.

### Study selection and data extraction

On completion of searches, we used Endnote version 21 (Clarivate, Philadelphia, PA, USA) and Covidence (Melbourne, Australia) software to identify and remove duplicates. Two reviewers then independently screened titles and abstracts and excluded obviously irrelevant citations. Subsequently, two reviewers reviewed the full-text of potentially eligible studies. At each stage, disagreements were resolved through consensus.

Data were extracted by a single reviewer and checked for accuracy by a second reviewer using a piloted data extraction form. Key data extracted included study design, population, study setting, toxin and antidote studied, and relevant clinical outcomes.

### Risk of bias and certainty of evidence

Two reviewers independently evaluated risk of bias using an appropriate tool based on study design: The Cochrane risk-of-bias tool v 2.0 (RoB 2) for randomised controlled trials and ROBINS-I v 2 for non-randomised studies.^23^, ^24^ Disagreements were resolved by consensus, or discussion with a third reviewer. Risk of bias figures were generated using the robvis tool.^25^ For each outcome, toxin and intervention of interest, we assessed certainty of evidence using the GRADE system.^22^

### Strategy for data synthesis

We planned to analyse data separately for each toxicological cause of interest.

Our review protocol described our plan to assess eligible studies following data extraction for evidence of clinical, methodological, and statistical heterogeneity. Where studies were deemed to be sufficiently homogenous, we planned to undertake meta-analyses. In the event that meta-analysis was precluded by heterogeneity, we planned to present a narrative synthesis, reported in line with the Synthesis without meta-analysis (SWiM) guidance.^26^

Due to the available data in eligible studies, we report treatment effects as odds ratios with 95% confidence intervals. Where an odds ratio was not reported, we calculated it using available data wherever possible using Revman version 5.4 (Cochrane Collaboration, Copenhagen, Denmark).

Where data were reported, we planned two sub-group analyses for the primary outcome, namely cardiac arrest setting (in-hospital v out-of-hospital) and rhythm (shockable v non-shockable).

## Results

Our search identified 9,938 records from database searches, of which 8,526 remained after removal of duplicates and 90 remained after screening of titles and abstracts (Fig. 1). From database searches, we identified four eligible studies. A further 19 records were identified from citation tracking and our trial register search, of which none were eligible following screening. One study in children was excluded because the analysis combined both toxicological causes and metabolic abnormalities, with toxicological causes making up less than 5% of this cohort.^2^

**Fig. 1:**
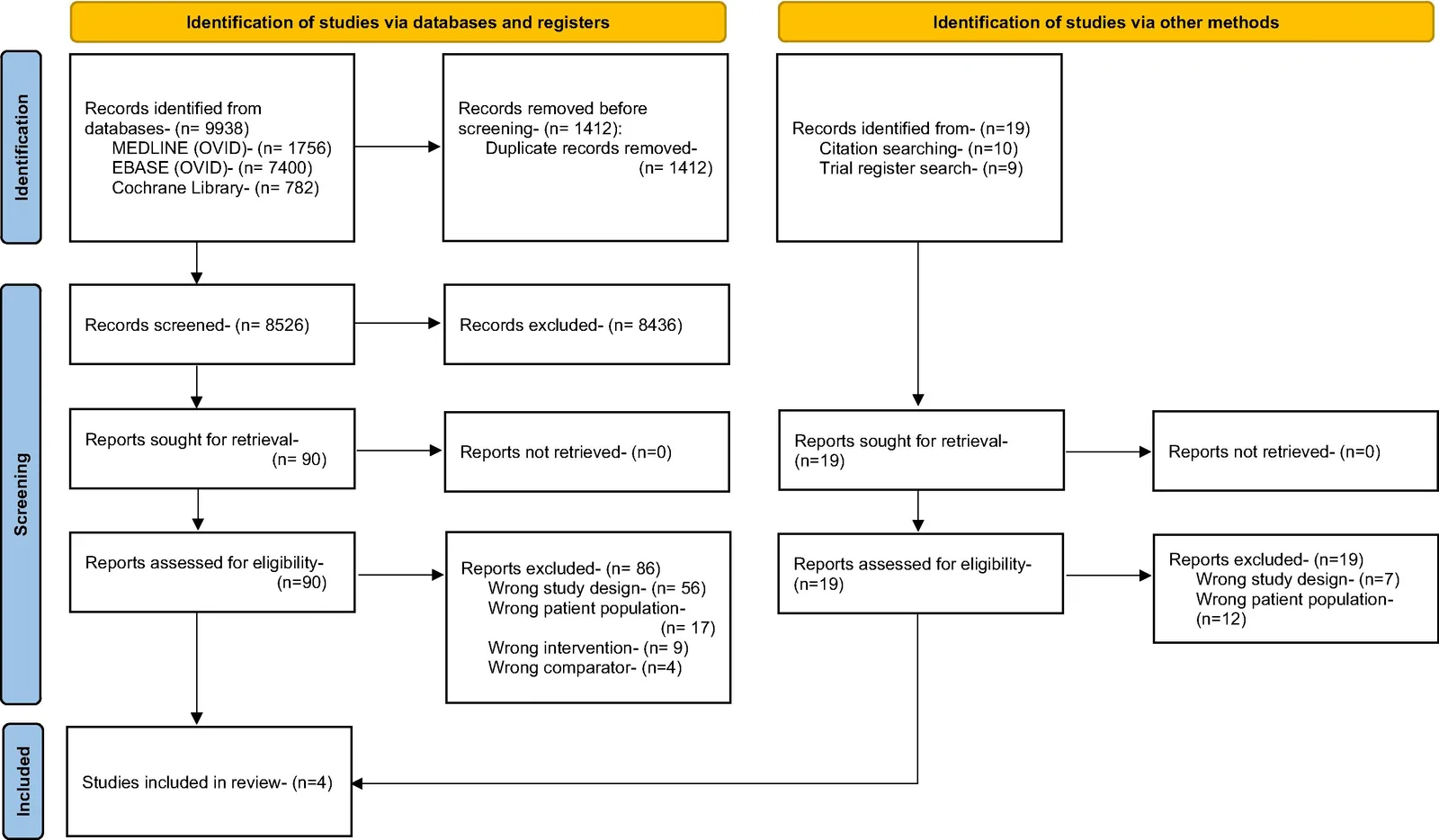
**Study identification flow diagram**.

Our four eligible studies were all retrospective cohort studies which enrolled between 10 and 1545 participants (Table 1; Supplementary Table S1).^27^, ^28^, ^29^, ^30^ Two studies used data from single emergency medical services (EMS) systems,^29^, ^30^ one study enrolled patients from across two hospitals,^28^ and the final study used data from a poisons service that covered three US states.^27^ One study included only patients in cardiac arrest^30^ and three studies reported patients in cardiac arrest as a sub-group of a larger cohort.^27^, ^28^, ^29^ One study included only adults.^30^ For the remaining three studies, one study provided patient-level data within a table, but we were unable to use data from children in the analysis as it was unclear what intervention they received.^29^ In a second study in which cardiac arrest patients were a sub-group of a larger cohort, it was reported that the age range in the whole cohort ranged from 14 to 89-years, but it is not known whether there were any children in the cardiac arrest sub-group.^27^ In the third study, where cardiac arrest patients were again a sub-group of a larger cohort, the mean age in the whole cohort was 48-years (standard deviation 17), such that it is likely that few, if any, children were included.^28^

**Table 1:** Study characteristics.

**Study**	**Study design**	**Setting (recruitment period)**	**Population**	**Intervention (s)**†	**Outcomes**
Alqahatani 2019	Retrospective cohort	Australian-EMS system (2000–2017)	1545 adult OHCA with suspected drug overdose	Sodium bicarbonate	Survival (hospital discharge)
Cole 2018‡	Retrospective cohort	USA-poison service covering three states (2000–2016)	41 cardiac arrests treated with high-dose insulin for beta-blocker or calcium channel-blocker poisoning	IV fat emulsion, Glucagon, Pacemaker, Methylene Blue, Intra-aortic balloon pump, ECMO	Survival (hospital discharge)
Fortin 2010‡	Retrospective cohort	France-EMS system (1995–2008)	61 OHCA (53 adults; 8 children) with smoke inhalation or cyanide salts ingestion treated with hydroxocobalamin	Single v two-dose hydroxocobalamin	ROSC, Survival (hospital discharge)
Masson 2012‡	Retrospective cohort	France-two hospitals (1999–2010)	10 persistent cardiac arrest following poisoning due to drug intoxication	E-CPR	Survival (ICU discharge)

Key: ECMO – extracorporeal membrane oxygenation; E-CPR – extracorporeal cardiopulmonary resuscitation; EMS – emergency medical service; ICU – intensive care unit; OHCA – out-of-hospital cardiac arrest.

† Compared with no intervention (unless stated).

‡ Cole 2018, Fortin 2010 and Masson 2012 reported a larger cohort incorporating both cardiac arrest and non-cardiac arrest patients – we report only the cardiac arrest cohort.

Two studies examined specific toxicological causes of cardiac arrest: smoke inhalation or cyanide salts^29^; and, beta-blockers or calcium-channel blockers.^27^ The remaining two studies included all toxicological causes of cardiac arrest.^28^, ^30^ Outcomes reported included ROSC (one study), survival at ICU discharge (one study), and survival at hospital discharge (three studies). No study reported survival with favourable neurological outcome or health-related quality-of-life.

### Risk of bias and certainty of evidence

Fig. 2 shows the ROBINS-I risk of bias results. All studies were found to be at critical risk of bias due to unmeasured confounding. Furthermore, three of the four studies reported only unadjusted analyses or patient-level data which allowed these analyses to be undertaken.^27^, ^28^, ^29^ The remaining study did adjust for some baseline characteristics, but did not take account of resuscitation time bias, which likely biased against the intervention group.^30^, ^31^

**Fig. 2:**
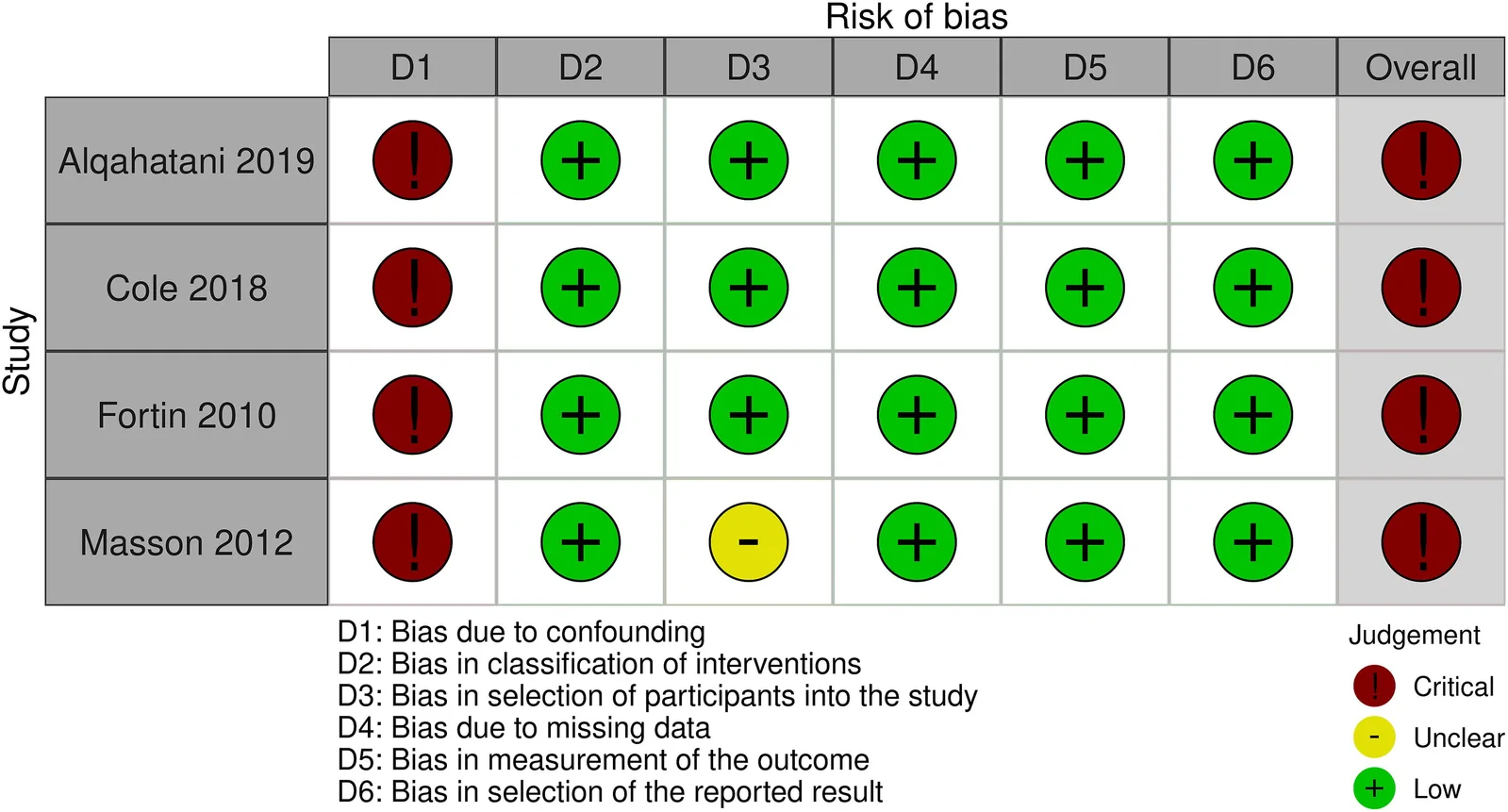
**Risk of bias assessment**.

For all interventions and associated outcomes, evidence certainty was assessed as very low (Supplementary Table S2). This predominantly reflected the observational study design and subsequent downgrading for extremely serious risk of bias and imprecision.

### General toxicological causes

Two studies explored general toxicological causes of cardiac arrest, with one evaluating the effect of sodium bicarbonate and one evaluating the effect of extracorporeal cardiopulmonary resuscitation (E-CPR).^28^, ^30^

#### Sodium bicarbonate

In a registry-based study of 1545 adults with out-of-hospital cardiac arrest in an Australian EMS system, the use of sodium bicarbonate was associated with lower odds of survival at hospital discharge (adjusted odds ratio (OR) 0.16, 95% CI 0.08–0.31; very low certainty evidence; Fig. 3).^30^

**Fig. 3:**
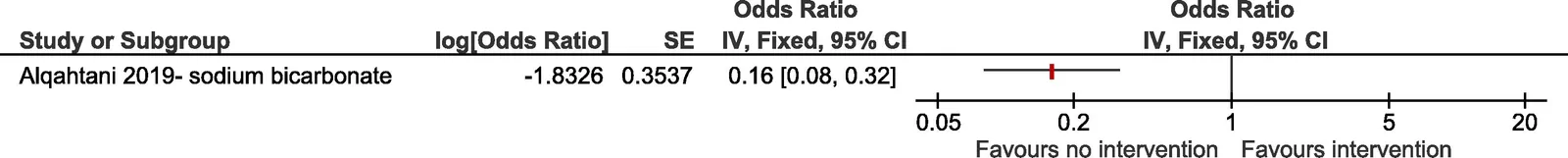
**Survival to discharge-studies reporting adjusted analyses**.

#### Extracorporeal CPR

For Extracorporeal Cardiopulmonary Resuscitation (ECPR), the single available study compared outcomes in 10 patients admitted to one hospital that provided ECPR (*n* = 3) with patients admitted to a second hospital that did not provide ECPR (*n* = 7). ECPR was associated with increased odds of survival at intensive care unit discharge (100% v 0%, unadjusted OR 105.00, 95% CI 1.71–6465.14; very low certainty evidence; Fig. 4).^28^

**Fig. 4:**
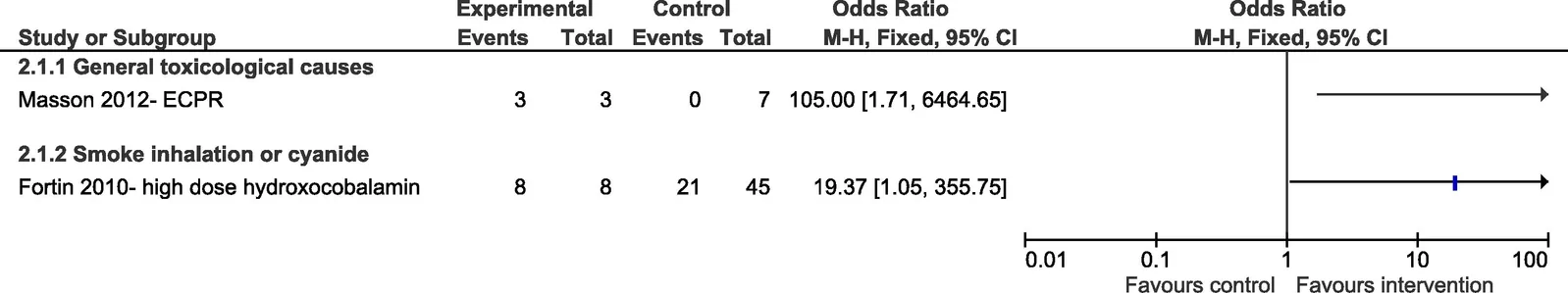
**Return of spontaneous circulation-studies reporting unadjusted analyses**. Masson 2012 reported ICU discharge. ECPR – extracorporeal cardiopulmonary resuscitation.

### Calcium-channel blockers and beta-blockers

#### Multiple interventions

Cole et al described a range of treatments provided to 61 adults with cardiac arrest following beta-blocker or calcium-channel blocker overdose, all of whom received high-dose insulin therapy.^27^ Additional interventions included intravenous lipid emulsion, glucagon, and methylene blue. It was uncertain whether all interventions were delivered during cardiac arrest or following ROSC. Across the six interventions evaluated, none was associated with a difference in survival to hospital discharge (all very low certainty evidence; Fig. 5).

**Fig. 5:**
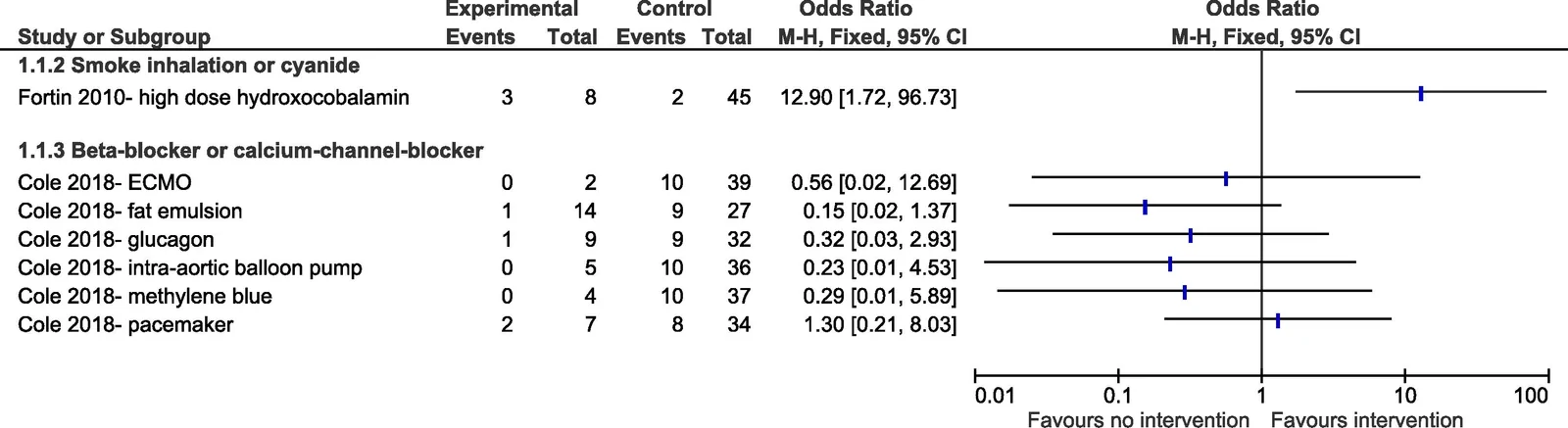
**Survival to hospital discharge-studies reporting unadjusted analyses**. ECMO – extracorporeal membrane oxygenation.

### Smoke inhalation and cyanide salts

#### Hydroxocobalamin

In a study of 61 patients (53 adults; 8 children) with cardiac arrest due to presumed cyanide poisoning from either smoke inhalation or cyanide salt ingestion and who received hydroxocobalamin, we used the reported patient-level data of adult patients which enabled us to compare outcomes in patients that received a single-dose (5 g) of hydroxocobalamin with those that received two doses (10 g) of hydroxocobalamin.^29^ Whilst the study included data on eight children, we were unable to use these as we were unable to determine whether they received one or two doses. The use of two doses of hydroxocobalamin, compared with one dose, was associated with improved rates of ROSC (OR 12.90; 95% CI 1.72–98.73; very low certainty evidence; Fig. 5) and survival to hospital discharge (OR 19.37; 95% CI 1.05–355.75; very low certainty evidence; Fig. 4).

### Sub-group analyses

Due to the nature of the available data, we were unable to undertake planned sub-group analyses.

## Discussion

Across four studies, we identified only very low certainty evidence for a range of interventions for different toxicological causes of cardiac arrest. These findings are challenging to interpret due to the high or critical risk of bias across index studies, particularly in the context of resuscitation time bias and very large reported treatment effects with wide confidence intervals.^31^ Whilst one study specifically included children, we were not able to extract any relevant paediatric data. Importantly, we did not find reliable evidence of harm in relation to any strategy.

The dearth of evidence in this area reflects the difficulties of conducting high-quality research on this patient population, due in part to the low incidence of cardiac arrest due to a toxicological cause and the wide range of drugs that may cause cardiac arrest.^1^, ^3^, ^5^ Furthermore, the incidence and specific toxicological causes of cardiac arrest may vary over time and by setting.^1^, ^3^, ^5^, ^30^, ^32^

Our review builds on a range of previous toxicological guidelines which describe treatment following a presumed severe poisoning, including those developed by the AHA in 2023 and updated in 2025.^17^, ^18^ The AHA guidelines describe recommended treatment strategies across 13 drug classes for patients both at risk of cardiac arrest and in cardiac arrest. In contrast to the American Heart Association statement, our review specifically focussed on intra-arrest treatment and we chose not to use indirect evidence (e.g. animal studies, ex vivo studies, or extrapolate evidence from non-cardiac arrest populations), very low quality evidence (e.g. case reports), or expert opinion. This decision reflects both the poor generalisability from animal studies to human cardiac arrest, and the high risk of reporting bias in case reports.^33^, ^34^, ^35^ In so doing, we highlight the paucity of evidence to support modifications to Advanced Life Support in patients with a presumed toxicological cause of cardiac arrest.

In deciding whether to modify Advanced Life Support algorithms to administer potential antidotes during cardiac arrest, providers may seek to draw on indirect evidence from patients who are not in cardiac arrest. Whilst this may represent reasonable decision-making in the face of severely limited evidence, it may be problematic for three reasons. Firstly, it can be difficult to determine what substance caused the cardiac arrest, particularly given that it is common for patients to take multiple drugs and require urgent treatment before a detailed collateral history can be obtained.^5^ Secondly, even when an antidote is in common use outside of cardiac arrest, evidence underpinning its use might still be very limited. For example, whilst glucagon is often cited as a potential antidote for beta-blocker toxicity, supporting evidence is seemingly predominantly limited to case series and a single trial of healthy volunteers.^17^, ^18^, ^36^ Third, for treatments to be effective in cardiac arrest, they must rapidly address the underlying cause of cardiac arrest to minimise time to ROSC. Available evidence supports the digoxin-specific fab fragments in the treatment of oleander and digoxin poisoning in adults and children who are not in cardiac arrest, but its delayed onset of action may make it less useful during cardiac arrest.^37^, ^38^, ^39^ In a study in which adults who were not in cardiac arrest received digoxin-specific fab fragments over 20–30 min, median time to initial response was 19 min (interquartile range 0–60 min).^39^ The ability of an antidote or enhanced elimination technique to actually reverse the poisoning before irreversible brain and other organ injury occurs should not be presumed without evidence. Fourth, while toxins are listed as a “reversible” cause of cardiac arrest, this effect relies in some cases on renal elimination or hepatic metabolism of the toxin, which may be prolonged and dependent on blood flow. Some patients have survived toxin-induced cardiac arrest with E-CPR^40^ or prolonged CPR.^41^, ^42^, ^43^

Our review has several important limitations. First, we included only human comparative studies of intra-arrest strategies. Whilst we provide our rationale for this approach above, it is possible that the use of indirect evidence, such as animal studies, or case reports might have provided additional insights. Secondly, we found no usable evidence in children. Thirdly, within index studies, the timing of intervention delivery was often poorly described, such that it was not always clear whether they were administered during cardiac arrest.

In conclusion, current evidence does not support the routine modification of standard advanced life support in the context of a cardiac arrest due to a presumed toxicological cause in adults or children. Whilst further research is needed, this will likely be challenging to undertake.

## CRediT authorship contribution statement

**Keith Couper:** Writing – review & editing, Writing – original draft, Supervision, Project administration, Methodology, Investigation, Formal analysis, Conceptualization. **Amani Alenazi:** Writing – review & editing, Investigation. **James Gray:** Writing – review & editing, Investigation. **Brian Grunau:** Writing – review & editing, Methodology, Investigation, Conceptualization. **Stephan Katzenschlager:** Writing – review & editing, Methodology, Investigation, Conceptualization. **Ben Rutherford:** Writing – review & editing, Investigation. **Michael D. Simpson:** Writing – review & editing, Investigation. **Ian R. Drennan:** Writing – review & editing, Methodology, Conceptualization. **Barney Scholefield:** Writing – review & editing, Methodology, Conceptualization. **Eric J. Lavonas:** Writing – review & editing, Methodology, Investigation, Conceptualization.

## Funding

This systematic review was commissioned by ILCOR. The work of ILCOR is underpinned by contributions from its member councils. None of the authors received payment from this funding source to complete this systematic review.

KC is supported by Resuscitation Council UK as director of Resuscitation Outcomes Consortium-UK. MDS was supported by National Heart, Lung, and Blood Institute (T32HL155020).

## Members of the ILCOR Adult Advanced Life Support Task Force and ILCOR Pediatric Life Support Task Force

Anders Aneman, Bernd W. Böttiger, Yew Woon Chia, Conor Crowley, Sonia D’Arrigo, Charles Deakin, Shannon M. Fernando, Asger Granfeldt, Karen Hirsch, Mathias J. Holmberg, Peter J. Kudenchuk, Carrie Leong, Ari Moskowitz, Robert W. Neumar, Tonia Nicholson, Nikolaos Nikoloau, Jerry P. Nolan, Shinichiro Ohshimo, Brian J. O’Neil, Michael Parr, Sarah M. Perman, Helen Pocock, Claudio Sandroni, Tommaso Scquizzato, Markus B. Skrifvars, Jasmeet Soar, Neville Vlok, Michelle Welsford, Johannes Wittig, Carolyn M. Zelop, Jason Acworth, Lokesh Kumar Tiwari, Tia T. Raymond, Andrea Christoff, Arun Bansal, Alexis Topjian, Monica Kleinman, Hiroshi Kurosawa, Michelle Myburgh, Jimena del Castillo, Joseph Rossano, Ryan W. Morgan, Leandra Rech, Anne-Marie Guerguerian, Vinay Nadkarni, Thomaz Bittencourt Couto, Stephen M. Schexnayder, Gabrielle Nuthall, Janice A. Tijssen, Gene Yong-Kwang Ong, Ester Ambunda, Laurie J. Morrison, Dianne L. Atkins, Allan R. de Caen

## Declaration of competing interest

KC is an associate editor and BG is an editorial board member of Resuscitation Plus. These individuals were not involved in the peer review or editorial decision-making for this manuscript. The remaining authors declare no conflicts of interest.
