# Supplementary material for: Intra-arrest strategies to treat cardiac arrest due to a non-opioid toxicological causes in adults and children: a systematic review
Source: Resusc Plus. 2026 Sep 24;32:101501. doi: 10.1016/j.resplu.2026.101501 (PMC13659757; doi:10.1016/j.resplu.2026.101501)
Supplement: Supplementary file 1 [file mmc1.pdf]

Intra-arrest strategies to treat cardiac arrest due to a non-opioid toxicological causes in adults and children: a systematic review

Supplementary materials

ILCOR Review Template 2

Specific toxins of interest 12

Summary of searches 13

MEDLINE search 13

- Original search 13
- MEDLINE search update 17

EMBASE search 22

- Original search 22
- EMBASE search update 26

Cochrane library search 30

- Cochrane search block one (original search) 30
- Cochrane search block two (original search) 33
- Cochrane search block three (original search) 35
- Cochrane search block four (original search) 37
- Cochrane updated search 38

World Health Organization International Clinical Trials Registry Platform 39

ClinicalTrials.gov 39

Table S1: further information on study populations 40

Table S2: GRADE evidence table 41

V 4.0 PICOST Template 11 March 2019 CEE approved

This template is designed to help guide the preparation of a research question by population intervention comparator outcome study design and timeline (PICOST) to guide a systematic review. This is the most important step in the design of a systematic review (SR). The PICOST is prepared by the task force, approved by the continuous evidence evaluation committee (CEE), edited by the expert systematic reviewer or the knowledge synthesis lead and forwarded by CEE to ILCOR executive and the task force chair(s) for acknowledgement. This acknowledgement determines the time zero or start of the SR workflow.

One of the main roles of the CEE WG is to facilitate the work being performed by the various Task Forces. The CEE WG recognizes and greatly appreciates the content expertise of each Task Force. Our role is neither to create PICOSTS nor to challenge the Task Force recommendations regarding the importance of a given PICOST. However, to ensure efficiency, and fulfill our mandate, the CEE WG requires that submitted PICOSTS are designed to answer the question that was intended by the submitting Task Force. Detailed attention to the content of submitted PICOSTs is a prerequisite for the CEE WG to help fulfill its mandate of assigning the appropriate ILCOR-AHA resources for each PICOST. This helps ensure that resources are distributed appropriately with the intention of maximizing efficiency and productivity. Task Forces are encouraged to provide the rationale for each proposed PICOST in the 'background' section of the proposal as a proactive means of minimizing interpretation issues during review by the CEE WG.

`

PICOST Short Title *(edit)*	**Interventions to treat toxicological causes of cardiac arrest**

1. Research Question based on PICOST

(Population, Intervention, Control, Outcomes, Study design and Timeframe)

PICOST	Description *(with recommended text)*
**Population**	Adults in any setting (in-hospital or out-of-hospital) with cardiac arrest due to a presumed toxicological cause
**Intervention**	Any specific alteration to standard Advanced Life Support during ongoing cardiopulmonary resuscitation
**Comparison**	Either:<br>1) Standard Advanced Life Support, OR,<br>2) Any other specific alteration to standard Advanced Life Support during ongoing cardiopulmonary resuscitation
**Outcomes**	Health-related quality of life at 3-months/ 6-months (or longest follow-up)- Critical<br>Survival with favourable neurological outcome at discharge/30-days; 3-months; 6-months (or longest follow-up)- Critical<br>Survival at discharge/30-days; 3-months; 6-months (or longest follow-up)- Critical<br>Sustained return of spontaneous circulation (at hospital handover or 20-minutes)-Important<br>Any return of spontaneous circulation- Important
**Study Design**	Randomized controlled trials (RCTs) and non-randomized studies with a control group (e.g. non-randomized controlled trials, interrupted time series, controlled before-and-after studies, cohort studies) in humans.<br><br>Unpublished studies (e.g., conference abstracts, trial protocols) and non-human studies (e.g. animal, simulation) are excluded.
**Timeframe**	All years and all languages are included as long as there is an English abstract

2. ILCOR Priority Team

Single TF PICOST

***TF chair or delegate will confirm COI through up to date disclosures prior to assignment.**
^ CEE chair or delegate will confirm COI through up to date disclosures prior to accepting assignment.
ILCOR COI Policy and the COI Committee are resources to address any questions.

Role	Name	Notes
*Lead Task Force Content Experts (1/2):		(preferably TF members*)
*Lead Task Force Content Experts (2/2):		(preferably TF members*)
*Lead Task Force Content Expert Mentee (1)		(**ESR assigned PICOST only**, preferably TF members*)
^KSU or ESR	CEE assigned	(assigned by CEE)
^ESR Mentee (1)	CEE assigned	(assigned by CEE from roster **ESR assigned PICOST only**)
^Domain Lead (1):	CEE assigned	(assigned by CEE)

^CEE WG representative (1)	CEE assigned	(assigned by CEE)

Nodal TF PICOST

Role	Name	Notes
*Lead Task Force Content Experts (1/2):	Keith Couper	(preferably TF members*)
*Lead Task Force Content Experts (2/2):	Brian Grunau/ Eric Lavonas/ Michael Simpson	(preferably TF members*)
*Lead Task Force Content Expert Mentee (1)		(**ESR assigned PICOST only**, preferably TF members*)
*Nodal TF Content Expert(s)	Stephan Katzenschlager/ Barney Scholefield	(when more than one TF involved, 1 per nodal TF): (preferably TF members*)
^KSU or ESR (1)	CEE assigned	(assigned by CEE)
^ESR Mentee (1)	CEE assigned	(assigned by CEE from roster, **ESR assigned PICOST only**)
^Domain Leads (1):	CEE assigned	(assigned by CEE)
^CEE WG representative (1)	CEE assigned	(assigned by CEE)

* non TF members must submit COI documentation and be approved prior to assignment

Back up Content Experts (Optional)

Role	Name	Notes
*Back up PICOST content expert	Optional	(preferably TF members*)
*Back up content expert (Nodal TF)		(preferably TF members*)

Back Up Content Expert recommended for Lead and Nodal task forces but not mandated
Back up Content Experts (1 per TF): (content experts who step in if a content expert becomes unable to complete the work. They are not on the team, nor eligible for authorship unless they are asked by TF chair to step into the role)

3. Active and Reposed PICOs Related to scope of work for this PICOST:

Insert all PICOs as worded on the master document and include AHA number
Please add the categorization and prioritization ranking by lead TF and nodal TF of the PICOs listed above (note: This information is available in the file: ILCOR PICO List on ilcor.org).

This new PICOST replaces the following existing PICOSTs		
**TCA**	Tricyclic antidepressant toxicity	3450
**Digoxin**	Digoxin toxicity	3452
**Cyanide**	Cyanide Toxicity	3453
**Cocaine**	Cocaine Toxicity	3454
**Carbon Monoxide**	Carbon monoxide Toxicity	3455
**CCBs**	Calcium channel blocker Toxicity	3456
**BBs**	Beta blocker Toxicity	3457
**Benzodiazepines**	Benzodiazepine Toxicity	3458
**Lipid Emulsion Therapy**	Lipid therapy for cardiac arrest	3459

	secondary to drug toxicity	
**Sodium Channel Blockers**	Sodium Channel Blockers	3460
**Inhalants/Hydrocarbons**	Inhalants/hydrocarbons	3461
**Organophosphates**		3462
**Methylene Blue**	methylene blue for cardiac arrest due to nitrate poisoning	3463

Opioid toxicity remains as a self-standing PICOST.

4. **Definitions:** *(This should include definitions of all the relevant terms identified in the PICOST and in the body of literature related to this topic identified during task force discussion)*

Cardiac arrest due to toxicological cause – a cardiac arrest where the known or presumed cause is the ingestion, inhalation or injection of a substance, whether that is deliberate or accidental

5. **Background and Rational for this PICOST:** *(Why is this SR important to complete now and what are the potential clinical implications of completing this review? Include how this new science is anticipated to impact on the existing ILCOR recommendations. References required as per ILCOR format embedded in text (last name first author, year of publication, first page number and list full references at bottom of form).*

Data from the CARES registry suggests that up to 8% OHCA are attributable to a drug overdose (Shekhar 2024), although an important proportion of these are likely to be associated with opioids. Individuals that have a cardiac arrest associated with a drug overdose tend to be younger, have fewer co-morbidities, and may experience better outcomes.

For some drugs, there is a known treatment strategy (including specific antidote). However, it is often unclear whether the administration of these strategies in cardiac arrest is clinically effective. For example, in ILCOR's recent review on opioid toxicity, contradictory evidence was found on specific modification to standard Advanced Life Support (Grunau 2025).

As such, there is a need to summarise evidence on Advanced Life Support treatment modifications in cardiac arrest due to a toxicological cause.

6. **Notes:** *(the nuances and subtleties of the task force discussion; it is important to include anything that doesn't fit in any other PICOST section but the task force feels this information is contributory to the question)* If it is anticipated by CEs and task force that there will be insufficient direct evidence, and indirect evidence will be used to answer the question the CE or Taskforce needs to document clearly what they mean by indirect and confirm indirect evidence exists.

This new PICOST will replace a number of existing PICOSTs. The opioid toxicity PICOST will remain as a separate PICOST.

Specific toxicological causes of interest are: tricyclic antidepressant, digoxin/ cardiac glycosides, cyanide, cocaine, carbon monoxide, calcium channel blocker, beta-blocker, benzodiazepine, local anaesthetic, methemoglobin formers, sodium channel blocker, chloroquine/ hydroxychloroquine, selective serotonin re-uptake inhibitors, loperamide, bupropion, inhalants/ hydrocarbons, organophosphates, jellyfish envenomation, hydrofluoric acid/

fluoride.

"Beta blockers" are synonymous with beta-adrenergic receptor antagonists.

"Calcium channel blockers" are synonymous with L-type calcium channel antagonists.

The drugs of interest were based on previous PICOSTs and drugs that have emerged as being of potential interest over recent years.

Each drug/ group of drugs will be analysed separately.

7. **Task Force Suggested Outcomes:** *(These will be updated/modified after the SR search is performed and the total number of critical or important outcomes should be no more than 7)*

Health-related quality of life at 3-months/ 6-months (or longest follow-up)- Critical
Survival with favourable neurological outcome at discharge/30-days; 3-months; 6-months (or longest follow-up)- Critical
Survival at discharge/30-days; 3-months; 6-months (or longest follow-up)- Critical
Sustained return of spontaneous circulation (at hospital handover or 20-minutes)- Important
Any return of spontaneous circulation- Important

8. **Key recent studies:** (*sentinel papers that are appropriate to answer this PICO. Please insert full references)*

Elmer J, Lynch MJ, Kristan J, Morgan P, Gerstel SJ, Callaway CW, Rittenberger JC; Pittsburgh Post-Cardiac Arrest Service. Recreational drug overdose-related cardiac arrests: break on through to the other side. Resuscitation. 2015 Apr;89:177-81.

Shekhar AC, Nathanson BH, Mader TJ, Coute RA. Cardiac Arrest Following Drug Overdose in the United States: An Analysis of the Cardiac Arrest Registry to Enhance Survival. J Am Heart Assoc. 2024 Feb 6;13(3):e031245. doi: 10.1161/JAHA.123.031245. Epub 2024 Jan 31. PMID: 38293840; PMCID: PMC11056133.

9. **Recent systematic reviews:** *(directly or indirectly addressing this PICO. Please insert full references)*

Grunau B, O'Neil BJ, Giustini D, Drennan IR, Lavonas EJ; Advanced Life Support Task Force of the International Liaison Committee on Resuscitation. Opioid-associated cardiac arrest: A systematic review of intra-arrest naloxone and other opioid-specific advanced life-support therapies. Resusc Plus. 2025 Feb 14;22:100906. doi: 10.1016/j.resplu.2025.100906. PMID: 40129502; PMCID: PMC11930592.

ILCOR review covering opioids (not covered by this review)

10. Review for ongoing clinical trials or unpublished work *(Use recommend links below)*:

1. International Clinical Trials Registry Platform (www.who.int/ictrp/en/)
2. US clinical trials registry (www.clinicaltrials.gov)
3. Cochrane CENTRAL (http://www.cochranelibrary.com/about/central-landing-page.html)

None identified

11. List *A priori* Subgroup analyses: *(defined a priori based on expert opinion. Note: number of comparator tables in systematic review = no. of outcomes x no. of comparison x no. of subgroup, consider focusing **absolute essential subgroups only**. If paediatrics or neonatal TF are involved a neonatal and/or a paediatrics specific subgroup analysis is required*).

We will only undertake sub-group analyses for the review primary outcome (survival at 30-days/ discharge).
- IHCA v OHCA
- Shockable v non-shockable rhythm
- Adults v children

12. Anticipated Workload *(required to guide volume of work estimate for ESR/KSU allocation):*

Approximate number of abstracts to screen based on published SRs or prior ILCOR work	**N= 2000 based on MEDLINE search (assume 5000 across MEDLINE/ EMBASE/ Cochrane)**
Approximate number of full manuscripts to review based on published SRs or prior ILCOR work	**N= Unknown**

13. Target Peer Reviewed Journals for SR Publication **(rank by priority if more than one)**

1. First choice journal	**Resuscitation**
2. Second choice	**Resuscitation Plus**
3. Third choice	*Journal of Medical Toxicology*

14. References (list references cited by author, year, first page in the Background and Rational)

Elmer J, Lynch MJ, Kristan J, Morgan P, Gerstel SJ, Callaway CW, Rittenberger JC; Pittsburgh Post-Cardiac Arrest Service. Recreational drug overdose-related cardiac arrests: break on through to the other side. Resuscitation. 2015 Apr;89:177-81.

Grunau B, O'Neil BJ, Giustini D, Drennan IR, Lavonas EJ; Advanced Life Support Task Force of the International Liaison Committee on Resuscitation. Opioid-associated cardiac arrest: A systematic review of intra-arrest naloxone and other opioid-specific advanced life-support therapies. Resusc Plus. 2025 Feb 14;22:100906. doi: 10.1016/j.resplu.2025.100906. PMID: 40129502; PMCID: PMC11930592.

Shekhar AC, Nathanson BH, Mader TJ, Coute RA. Cardiac Arrest Following Drug Overdose in the United States: An

Analysis of the Cardiac Arrest Registry to Enhance Survival. J Am Heart Assoc. 2024 Feb 6;13(3):e031245. doi: 10.1161/JAHA.123.031245. Epub 2024 Jan 31. PMID: 38293840; PMCID: PMC11056133.

15. Confirmation of approval steps (completed by CEE)

Steps	Insert Date (day/month/year)
Submission to CEE WG	Completed by CEE
Approved by CEE WG	Completed by CEE
Acknowledged by ILCOR Board	Completed by CEE
Acknowledged by task force(s)	Completed by CEE
Submission to SMH IS	Completed by CEE

Planned search strategy

Ovid MEDLINE(R) ALL

1. exp Heart Arrest/
2. Ventricular Fibrillation/
3. Resuscitation/
4. Heart Massage/
5. exp Cardiopulmonary Resuscitation/
6. cardi* arrest*.tw,kf.
7. heart arrest*.tw,kf.
8. OHCA.tw,kf.
9. IHCA.tw,kf.
10. CPR.tw,kf.
11. advanced cardiac life support.tw,kf.
12. ACLS.tw,kf.
13. basic life support.tw,kf.
14. BLS.tw,kf.
15. asystol*.tw,kf.
16. pulseless electrical activity.tw,kf.
17. pulseless ventricular tachycardia.tw,kf.
18. (return of circulation or return of spontaneous circulation or ROSC).tw,kf.
19. ventricular fibrillation*.tw,kf.
20. chest compression*.tw,kf.
21. 1 or 2 or 3 or 4 or 5 or 6 or 7 or 8 or 9 or 10 or 11 or 12 or 13 or 14 or 15 or 16 or 17 or 18 or 19 or 20
22. benzodiazepines/ or alprazolam/ or benzodiazepinones/ or chlordiazepoxide/ or clobazam/ or clorazepate dipotassium/ or estazolam/ or medazepam/ or midazolam/ or olanzapine/ or triazolam/ or diltiazem/
23. (benzodiazepine* or alprazolam or bromazepam or clonazepam or diazepam or flumazenil or flunitrazepam or flurazepam or lorazepam or nitrazepam or oxazepam or temazepam or chlordiazepoxide or clobazam or clorazepate or estazolam or midazolam or nayzilam or triazolam).mp. [mp=title, book title, abstract, original title, name of substance word, subject heading word, floating sub-heading word, keyword heading word, organism supplementary concept word, protocol supplementary concept word, rare disease supplementary

concept word, unique identifier, synonyms, population supplementary concept word, anatomy supplementary concept word]
24. Adrenergic beta-Antagonists/
25. (beta block* or acebutolol or atenolol or bisoprolol or metoprolol or nadolol or nebivolol or propranolol or sotalol or betaxolol or pindolol or alprenolol or timolol).mp. [mp=title, book title, abstract, original title, name of substance word, subject heading word, floating sub-heading word, keyword heading word, organism supplementary concept word, protocol supplementary concept word, rare disease supplementary concept word, unique identifier, synonyms, population supplementary concept word, anatomy supplementary concept word]
26. calcium channel agonists/ or calcium channel blockers/ or amlodipine/ or amlodipine besylate, olmesartan medoxomil drug combination/ or amrinone/ or bencyclane/ or bepridil/ or carvedilol/ or cinnarizine/ or conotoxins/ or diltiazem/ or felodipine/ or fendiline/ or flunarizine/ or gallopamil/ or isradipine/ or lamotrigine/ or lidoflazine/ or mibefradil/ or nicardipine/ or nifedipine/ or nimodipine/ or nisoldipine/ or nitrendipine/ or perhexiline/ or prenylamine/ or risedronic acid/ or tiapamil hydrochloride/ or verapamil/
27. (calcium channel block* or calcium channel antag* or amlodipine or diltiazem or felodipine or isradipine or nicardipine or nifedipine or verapamil).mp. [mp=title, book title, abstract, original title, name of substance word, subject heading word, floating sub-heading word, keyword heading word, organism supplementary concept word, protocol supplementary concept word, rare disease supplementary concept word, unique identifier, synonyms, population supplementary concept word, anatomy supplementary concept word]
28. cocaine/ or crack cocaine/
29. cocaine.mp.
30. Cyanides/
31. Nitroprusside/ae, po, to [Adverse Effects, Poisoning, Toxicity]
32. cobamides/ or hydroxocobalamin/
33. Nitrites/
34. (cyanide or nitroprusside or nitrite or hydroxocobalamin or cobinamide).mp.
35. oleander.mp. or Nerium/
36. Bufanolides/
37. (bufadeinolide or cardenolide).mp.
38. lily of the valley.mp. or Convallaria/
39. foxglove.mp. or Digitalis/
40. Immunoglobulin Fab Fragments/
41. (digibind or digifab or digitab).mp.
42. anesthetics, local/ or benzocaine/ or bupivacaine/ or carticaine/ or dibucaine/ or etidocaine/ or levobupivacaine/ or lidocaine/ or lidocaine, prilocaine drug combination/ or mepivacaine/ or prilocaine/ or procaine/ or propoxycaine/ or ropivacaine/ or tetracaine/ or trimecaine/
43. (local anesthet* or benzocaine or bupivacaine or carbocaine or chloroprocaine or lidocaine or mepivacaine or procaine or prilocaine or proparacaine or ropivacaine).mp.
44. Nerve Block/ae [Adverse Effects]
45. Anesthesia, Dental/ae, co [Adverse Effects, Complications]
46. bier* block.mp.
47. Fat Emulsions, Intravenous/
48. (lipid emulsion* or intralipid or lipid rescue).mp.
49. Soybean Oil/tu [Therapeutic Use]
50. Methemoglobinemia/
51. (methemoglobin* or methaemoglobin*).mp.
52. Organophosphate Poisoning/
53. Organophosphates/ae, to [Adverse Effects, Toxicity]
54. organophosphorus compounds/ or organophosphates/ or carbamyl phosphate/ or chlorfenvinphos/ or dichlorvos/ or mevinphos/ or monocrotophos/ or naled/ or organothiophosphates/ or amifostine/ or chlorpyrifos/ or coumaphos/ or cystaphos/ or diazinon/ or dimethoate/ or disulfoton/ or echothiophate iodide/ or fenitrothion/ or fenthion/ or malathion/ or parathion/ or methyl parathion/ or phorate/ or phosmet/ or temefos/ or paraoxon/ or phosphamidon/ or tritolyl phosphates/ or aminoethylphosphonic acid/ or armin/ or cidofovir/ or diphosphonates/ or organofluorophosphonates/ or isoflurophate/ or sarin/ or soman/ or

organothiophosphonates/ or fonofos/ or leptophos/ or "phenylphosphonothioic acid, 2-ethyl 2-(4-nitrophenyl) ester"/ or azinphosmethyl/
55. Nerve Agents/
56. (ortanophosphate* or organothiophosphate* or organophosphorous* or nerve agent* or nerve gas*).mp.
57. (carbamyl* or chlorfenvinphos or dichlorvos or mevinphos or amifostine or chlorpyrifos or coumaphos or diazinon or fenitrothion or fenthion or malathion).mp. or *parathion/ or phorate.mp. or phosmet.mp. or temefos.mp. or paraxon*.mp. or phosphamidon.mp. or cidofovir.mp. or isoflurophate.mp. or sarin.mp. or soman.mp. or vx.mp. or novichok.mp.
58. carbamates/ or aldicarb/ or benomyl/ or carbamyl phosphate/ or carbaryl/
59. oximes/ or obidoxime chloride/ or pralidoxime compounds/ or trimedoxime/
60. (2-pam or 2pam or protopam or pralidoxime or HI-6 or asoxime).mp.
61. obidoxime.mp.
62. Antidepressive Agents, Tricyclic/
63. (tricyclic antidepress* or cyclic antidepress*).mp.
64. amitriptyline/ or nortriptyline/ or protriptyline/
65. Amoxapine/
66. clomipramine/ or desipramine/ or imipramine/ or lofepramine/ or mianserin/ or trimipramine/
67. Doxepin/
68. Maprotiline/
69. Sodium Channel Blockers/
70. (sodium channel block* or sodium channel antag*).mp.
71. (bicyclic antidepressant* or tetracyclic antidepressant*).mp.
72. (amitriptyline or amoxapine or desipramine or doxepin or imipramine or nortriptyline or protriptyline or trimipramine or maprotiline or carbamazepine or chloroquine or diphenhydramine or flecainide or hydroxychloroquine or lamotrigine or lacosamide or propafenone or quinine or quinidine or thioridazine or taxus or yew or topiramate or venlafaxine or zonisamide or bupropion).mp.
73. Selective Serotonin Reuptake Inhibitors/
74. "Serotonin and Noradrenaline Reuptake Inhibitors"/ or Antidepressive Agents, Second-Generation/ or Antidepressive Agents/
75. (SSRI or SNRI or (Serotonin adj3 inhib*)).mp. [mp=title, book title, abstract, original title, name of substance word, subject heading word, floating sub-heading word, keyword heading word, organism supplementary concept word, protocol supplementary concept word, rare disease supplementary concept word, unique identifier, synonyms, population supplementary concept word, anatomy supplementary concept word]
76. (citalopram or escitalopram).mp. [mp=title, book title, abstract, original title, name of substance word, subject heading word, floating sub-heading word, keyword heading word, organism supplementary concept word, protocol supplementary concept word, rare disease supplementary concept word, unique identifier, synonyms, population supplementary concept word, anatomy supplementary concept word]
77. loperamide.mp.
78. lopermaide/ or citalopram/ or escitalopram/
79. sympathomimetics/ or amphetamine/ or benzphetamine/ or methamphetamine/ or methoxamine/
80. amphetamines/ or amphetamine/ or dextroamphetamine/ or 2,5-dimethoxy-4-methylamphetamine/ or p-hydroxyamphetamine/ or iofetamine/ or methamphetamine/ or 3,4-methylenedioxyamphetamine/ or n-methyl-3,4-methylenedioxyamphetamine/ or dimethoxyphenylethylamine/ or methoxamine/ or p-methoxy-n-methylphenethylamine/
81. excitatory amino acid agents/ or excitatory amino acid agonists/
82. Central Nervous System Stimulants/
83. *amphetamine/ or dexedrine.mp. or procentra.mp. or *cathinone/ or *methylpropion/ or methylone.mp. or pyrovalerone.mp. or methylphenidate.mp. or daytrana.mp. or concerta.mp. or jurnay.mp. or methylin.mp. or ritalin.mp.
84. p-chloroamphetamine/ or 2,5-dimethoxy-4-methylamphetamine/ or 3,4-methylenedioxyamphetamine/ or n-methyl-3,4-methylenedioxyamphetamine/
85. exp Substance-Related Disorders/co [complications]
86. exp Inhalant Abuse/co

87. exp Hydrocarbons, Halogenated/po
88. Fluorocarbons/ae, po, to
89. ((Volatile substance* or Inhalant* or Hydrocarbon* or flurocarbon*) adj2 ("use" or usag* or abus* misus* or inhal*)).ab,kf,ti.
90. ((sniff* or inhal* or volatile) adj4 (Sudden adj3 death)).ab,kf,ti.
91. Carbon Monoxide Poisoning/
92. Carbon monoxide/
93. carbon monoxide.mp.
94. hydrofluoric acid/
95. (hydrofluoric acid* or hydrogen fluorid*).mp. [mp=title, book title, abstract, original title, name of substance word, subject heading word, floating sub-heading word, keyword heading word, organism supplementary concept word, protocol supplementary concept word, rare disease supplementary concept word, unique identifier, synonyms, population supplementary concept word, anatomy supplementary concept word]
96. cnidaria/ or cubozoa/ or hydrozoa/ or scyphozoa/
97. Cnidarian venoms/
98. (jellyfish or jelly-fish or irukandji).mp. [mp=title, book title, abstract, original title, name of substance word, subject heading word, floating sub-heading word, keyword heading word, organism supplementary concept word, protocol supplementary concept word, rare disease supplementary concept word, unique identifier, synonyms, population supplementary concept word, anatomy supplementary concept word]
99. 22 or 23 or 24 or 25 or 26 or 27 or 28 or 29 or 30 or 31 or 32 or 33 or 34 or 35 or 36 or 37 or 38 or 39 or 40 or 41 or 42 or 43 or 44 or 45 or 46 or 47 or 48 or 49 or 50 or 51 or 52 or 53 or 54 or 55 or 56 or 57 or 58 or 59 or 60 or 61 or 62 or 63 or 64 or 65 or 66 or 67 or 68 or 69 or 70 or 71 or 72 or 73 or 74 or 75 or 76 or 77 or 78 or 79 or 80 or 81 or 82 or 83 or 84 or 85 or 86 or 87 or 88 or 89 or 90 or 91 or 92 or 93 or 94 or 95 or 96 or 97 or 98
100. drug overdose/ or prescription drug overuse/
101. Poisoning/
102. Toxi*.mp.
103. Poisons/
104. poison*.mp.
105. overdose*.mp.
106. Antidotes/
107. antidote*.mp.
108. "Drug-Related Side Effects and Adverse Reactions"/
109. intoxication.mp.
110. 100 or 101 or 102 or 103 or 104 or 105 or 106 or 107 or 108 or 109
111. 21 and 99 and 110
112. exp animals/ not humans.sh.
113. 111 not 112

Specific toxins of interest

Benzodiazepine
Beta-blocker
Bupropion
Calcium channel blockers
Carbon monoxide
Chloroquine/ hydroxychloroquine
Cocaine
Cyanide
Digoxin/ cardiac glycosides
Hydrofluoric acid/ fluoride
Inhalants/ hydrocarbons,
Jellyfish envenomation
Local anaesthetic
Loperamide
Methemoglobin formers
Organophosphates
Selective serotonin re-uptake inhibitors,
Sodium channel blocker
Tricyclic antidepressant

Summary of searches

	Original search		Updated search	
**Database/ source**	**Date**	**Hits**	**Date**	**Additional Hits**
MEDLINE	1st December 2025	1720	22nd July 2026	36
EMBASE	1st December 2025	6966	22nd July 2026	434
Cochrane Library	1st December 2025	697	22nd July 2026	85
Trial register: ClinicalTrials.gov	15th March 2026	1	22nd July 2026	0
Trial register: World Health Organization International Clinical Trials Registry Platform	15th March 2026	8	22nd July 2026	0

MEDLINE search

Original search

Ovid MEDLINE(R) <1946 to November Week 4 2025>
1 exp Heart Arrest/ 60374
2 Ventricular Fibrillation/ 18280
3 Resuscitation/ 29599
4 Heart Massage/ 3254
5 exp Cardiopulmonary Resuscitation/ 24584
6 cardi* arrest*.tw,kf. 47484
7 heart arrest*.tw,kf. 2521
8 OHCA.tw,kf. 4388
9 IHCA.tw,kf. 673
10 CPR.tw,kf. 15114
11 advanced cardiac life support.tw,kf. 1132
12 ACLS.tw,kf. 1224
13 basic life support.tw,kf. 2317
14 BLS.tw,kf. 2296
15 asystol*.tw,kf. 4446
16 pulseless electrical activity.tw,kf. 966
17 pulseless ventricular tachycardia.tw,kf. 365
18 (return of circulation or return of spontaneous circulation or ROSC).tw,kf. 5243
19 ventricular fibrillation*.tw,kf. 19739
20 chest compression*.tw,kf. 4464

21 1 or 2 or 3 or 4 or 5 or 6 or 7 or 8 or 9 or 10 or 11 or 12 or 13 or 14 or 15 or 16 or 17 or 18 or 19 or 20 138896

22 benzodiazepines/ or alprazolam/ or benzodiazepinones/ or chlordiazepoxide/ or clobazam/ or clorazepate dipotassium/ or estazolam/ or medazepam/ or midazolam/ or olanzapine/ or triazolam/ or diltiazem/ 52060

23 (benzodiazepine* or alprazolam or bromazepam or clonazepam or diazepam or flumazenil or flunitrazepam or flurazepam or lorazepam or nitrazepam or oxazepam or temazepam or chlordiazepoxide or clobazam or clorazepate or estazolam or midazolam or nayzilam or triazolam).mp. [mp=title, book title, abstract, original title, name of substance word, subject heading word, floating sub-heading word, keyword heading word, organism supplementary concept word, protocol supplementary concept word, rare disease supplementary concept word, unique identifier, synonyms, population supplementary concept word, anatomy supplementary concept word] 95384

24 Adrenergic beta-Antagonists/ 42730

25 (beta block* or acebutolol or atenolol or bisoprolol or metoprolol or nadolol or nebivolol or propranolol or sotalol or betaxolol or pindolol or alprenolol or timolol).mp. [mp=title, book title, abstract, original title, name of substance word, subject heading word, floating sub-heading word, keyword heading word, organism supplementary concept word, protocol supplementary concept word, rare disease supplementary concept word, unique identifier, synonyms, population supplementary concept word, anatomy supplementary concept word] 100081

26 calcium channel agonists/ or calcium channel blockers/ or amlodipine/ or amlodipine besylate, olmesartan medoxomil drug combination/ or amrinone/ or bencyclane/ or bepridil/ or carvedilol/ or cinnarizine/ or conotoxins/ or diltiazem/ or felodipine/ or fendiline/ or flunarizine/ or gallopamil/ or isradipine/ or lamotrigine/ or lidoflazine/ or mibefradil/ or nicardipine/ or nifedipine/ or nimodipine/ or nisoldipine/ or nitrendipine/ or perhexiline/ or prenylamine/ or risedronic acid/ or tiapamil hydrochloride/ or verapamil/ 85289

27 (calcium channel block* or calcium channel antag* or amlodipine or diltiazem or felodipine or isradipine or nicardipine or nifedipine or verapamil).mp. [mp=title, book title, abstract, original title, name of substance word, subject heading word, floating sub-heading word, keyword heading word, organism supplementary concept word, protocol supplementary concept word, rare disease supplementary concept word, unique identifier, synonyms, population supplementary concept word, anatomy supplementary concept word] 88069

28 cocaine/ or crack cocaine/ 27991

29 cocaine.mp. 45588

30 Cyanides/ 13111

31 Nitroprusside/ae, po, to [Adverse Effects, Poisoning, Toxicity] 499

32 cobamides/ or hydroxocobalamin/ 2106

33 Nitrites/ 21698

34 (cyanide or nitroprusside or nitrite or hydroxocobalamin or cobinamide).mp. 75000

35 oleander.mp. or Nerium/ 648

36 Bufanolides/ 1433

37 (bufadeinolide or cardenolide).mp. 493

38 lily of the valley.mp. or Convallaria/ 209

39 foxglove.mp. or Digitalis/ 3417
40 Immunoglobulin Fab Fragments/ 12581
41 (digibind or digifab or digitab).mp. 119
42 anesthetics, local/ or benzocaine/ or bupivacaine/ or carticaine/ or dibucaine/ or etidocaine/ or levobupivacaine/ or lidocaine/ or lidocaine, prilocaine drug combination/ or mepivacaine/ or prilocaine/ or procaine/ or propoxycaine/ or ropivacaine/ or tetracaine/ or trimecaine/ 72318
43 (local anesthet* or benzocaine or bupivacaine or carbocaine or chloroprocaine or lidocaine or mepivacaine or procaine or prilocaine or proparacaine or ropivacaine).mp. 76934
44 Nerve Block/ae [Adverse Effects] 3328
45 Anesthesia, Dental/ae, co [Adverse Effects, Complications] 1706
46 bier* block.mp. 190
47 Fat Emulsions, Intravenous/4386
48 (lipid emulsion* or intralipid or lipid rescue).mp. 5766
49 Soybean Oil/tu [Therapeutic Use] 188
50 Methemoglobinemia/ 3450
51 (methemoglobin* or methaemoglobin*).mp. 9318
52 Organophosphate Poisoning/ 2355
53 Organophosphates/ae, to [Adverse Effects, Toxicity] 1478
54 organophosphorus compounds/ or organophosphates/ or carbamyl phosphate/ or chlorfenvinphos/ or dichlorvos/ or mevinphos/ or monocrotophos/ or naled/ or organothiophosphates/ or amifostine/ or chlorpyrifos/ or coumaphos/ or cystaphos/ or diazinon/ or dimethoate/ or disulfoton/ or echothiophate iodide/ or fenitrothion/ or fenthion/ or malathion/ or parathion/ or methyl parathion/ or phorate/ or phosmet/ or temefos/ or paraoxon/ or phosphamidon/ or tritolyl phosphates/ or aminoethylphosphonic acid/ or armin/ or cidofovir/ or diphosphonates/ or organofluorophosphonates/ or isoflurophate/ or sarin/ or soman/ or organothiophosphonates/ or fonofos/ or leptophos/ or "phenylphosphonothioic acid, 2-ethyl 2-(4-nitrophenyl) ester"/ or azinphosmethyl/ 71761
55 Nerve Agents/ 537
56 (ortanophosphate* or organothiophosphate* or organophosphorous* or nerve agent* or nerve gas*).mp. 5065
57 (carbamyl* or chlorfenvinphos or dichlorvos or mevinphos or amifostine or chlorpyrifos or coumaphos or diazinon or fenitrothion or fenthion or malathion).mp. or *parathion/ or phorate.mp. or phosmet.mp. or temefos.mp. or paraxon*.mp. or phosphamidon.mp. or cidofovir.mp. or isoflurophate.mp. or sarin.mp. or soman.mp. or vx.mp. or novichok.mp. 35097
58 carbamates/ or aldicarb/ or benomyl/ or carbamyl phosphate/ or carbaryl/ 16854
59 oximes/ or obidoxime chloride/ or pralidoxime compounds/ or trimedoxime/ 10394
60 (2-pam or 2pam or protopam or pralidoxime or HI-6 or asoxime).mp. 2237
61 obidoxime.mp. 596
62 Antidepressive Agents, Tricyclic/ 10706
63 (tricyclic antidepress* or cyclic antidepress*).mp. 10061
64 amitriptyline/ or nortriptyline/ or protriptyline/ 8667

65 Amoxapine/ 342
66 clomipramine/ or desipramine/ or imipramine/ or lofepramine/ or mianserin/ or trimipramine/ 19610
67 Doxepin/ 871
68 Maprotiline/ 878
69 Sodium Channel Blockers/ 4552
70 (sodium channel block* or sodium channel antag*).mp. 7223
71 (bicyclic antidepressant* or tetracyclic antidepressant*).mp. 335
72 (amitriptyline or amoxapine or desipramine or doxepin or imipramine or nortriptyline or protriptyline or trimipramine or maprotiline or carbamazepine or chloroquine or diphenhydramine or flecainide or hydroxychloroquine or lamotrigine or lacosamide or propafenone or quinine or quinidine or thioridazine or taxus or yew or topiramate or venlafaxine or zonisamide or bupropion).mp. 130929
73 Selective Serotonin Reuptake Inhibitors/ 22094
74 "Serotonin and Noradrenaline Reuptake Inhibitors"/ or Antidepressive Agents, Second-Generation/ or Antidepressive Agents/ 59826
75 (SSRI or SNRI or (Serotonin adj3 inhib*)).mp. [mp=title, book title, abstract, original title, name of substance word, subject heading word, floating sub-heading word, keyword heading word, organism supplementary concept word, protocol supplementary concept word, rare disease supplementary concept word, unique identifier, synonyms, population supplementary concept word, anatomy supplementary concept word] 33106
76 (citalopram or escitalopram).mp. [mp=title, book title, abstract, original title, name of substance word, subject heading word, floating sub-heading word, keyword heading word, organism supplementary concept word, protocol supplementary concept word, rare disease supplementary concept word, unique identifier, synonyms, population supplementary concept word, anatomy supplementary concept word] 8455
77 loperamide.mp. 2255
78 lopermaide/ or citalopram/ or escitalopram/ 5746
79 sympathomimetics/ or amphetamine/ or benzphetamine/ or methamphetamine/ or methoxamine/ 35242
80 amphetamines/ or amphetamine/ or dextroamphetamine/ or 2,5-dimethoxy-4-methylamphetamine/ or p-hydroxyamphetamine/ or iofetamine/ or methamphetamine/ or 3,4-methylenedioxyamphetamine/ or n-methyl-3,4-methylenedioxyamphetamine/ or dimethoxyphenylethylamine/ or methoxamine/ or p-methoxy-n-methylphenethylamine/ 44671
81 excitatory amino acid agents/ or excitatory amino acid agonists/ 8426
82 Central Nervous System Stimulants/ 23162
83 *amphetamine/ or dexedrine.mp. or procentra.mp. or *cathinone/ or *methylpropion/ or methylone.mp. or pyrovalerone.mp. or methylphenidate.mp. or daytrana.mp. or concerta.mp. or jurnay.mp. or methylin.mp. or ritalin.mp. 17168
84 p-chloroamphetamine/ or 2,5-dimethoxy-4-methylamphetamine/ or 3,4-methylenedioxyamphetamine/ or n-methyl-3,4-methylenedioxyamphetamine/ 5740
85 exp Substance-Related Disorders/co [complications] 55437
86 exp Inhalant Abuse/co 82

87 exp Hydrocarbons, Halogenated/po 3140
88 Fluorocarbons/ae, po, to 3737
89 ((Volatile substance* or Inhalant* or Hydrocarbon* or flurocarbon*) adj2 ("use" or usag* or abus* misus* or inhal*)).ab,kf,ti. 4658
90 ((sniff* or inhal* or volatile) adj4 (Sudden adj3 death)).ab,kf,ti. 49
91 Carbon Monoxide Poisoning/ 5937
92 Carbon monoxide/ 19831
93 carbon monoxide.mp. 36962
94 hydrofluoric acid/ 1581
95 (hydrofluoric acid* or hydrogen fluorid*).mp. [mp=title, book title, abstract, original title, name of substance word, subject heading word, floating sub-heading word, keyword heading word, organism supplementary concept word, protocol supplementary concept word, rare disease supplementary concept word, unique identifier, synonyms, population supplementary concept word, anatomy supplementary concept word] 2865
96 cnidaria/ or cubozoa/ or hydrozoa/ or scyphozoa/ 5488
97 Cnidarian venoms/ 1903
98 (jellyfish or jelly-fish or irukandji).mp. [mp=title, book title, abstract, original title, name of substance word, subject heading word, floating sub-heading word, keyword heading word, organism supplementary concept word, protocol supplementary concept word, rare disease supplementary concept word, unique identifier, synonyms, population supplementary concept word, anatomy supplementary concept word] 2546
99 22 or 23 or 24 or 25 or 26 or 27 or 28 or 29 or 30 or 31 or 32 or 33 or 34 or 35 or 36 or 37 or 38 or 39 or 40 or 41 or 42 or 43 or 44 or 45 or 46 or 47 or 48 or 49 or 50 or 51 or 52 or 53 or 54 or 55 or 56 or 57 or 58 or 59 or 60 or 61 or 62 or 63 or 64 or 65 or 66 or 67 or 68 or 69 or 70 or 71 or 72 or 73 or 74 or 75 or 76 or 77 or 78 or 79 or 80 or 81 or 82 or 83 or 84 or 85 or 86 or 87 or 88 or 89 or 90 or 91 or 92 or 93 or 94 or 95 or 96 or 97 or 98 1011323
100 drug overdose/ or prescription drug overuse/ 16538
101 Poisoning/ 23271
102 Toxi*.mp. 1274185
103 Poisons/ 3464
104 poison*.mp. 165035
105 overdose*.mp. 31633
106 Antidotes/ 6690
107 antidote*.mp. 11081
108 "Drug-Related Side Effects and Adverse Reactions"/ 40977
109 intoxication.mp. 52001
110 100 or 101 or 102 or 103 or 104 or 105 or 106 or 107 or 108 or 109 1460568
111 21 and 99 and 110 2127
112 exp animals/ not humans.sh. 5396866
113 111 not 112 1720

MEDLINE search update

Ovid MEDLINE(R) <1946 to July Week 2 2026>

1 exp Heart Arrest/ 61495
2 Ventricular Fibrillation/ 18371
3 Resuscitation/ 29921
4 Heart Massage/ 3279
5 exp Cardiopulmonary Resuscitation/ 25213
6 cardi* arrest*.tw,kf. 49029
7 heart arrest*.tw,kf. 2557
8 OHCA.tw,kf. 4666
9 IHCA.tw,kf. 735
10 CPR.tw,kf. 15628
11 advanced cardiac life support.tw,kf. 1157
12 ACLS.tw,kf. 1258
13 basic life support.tw,kf. 2386
14 BLS.tw,kf. 2386
15 asystol*.tw,kf. 4517
16 pulseless electrical activity.tw,kf. 984
17 pulseless ventricular tachycardia.tw,kf. 374
18 (return of circulation or return of spontaneous circulation or ROSC).tw,kf. 5506
19 ventricular fibrillation*.tw,kf. 19939
20 chest compression*.tw,kf. 4592
21 1 or 2 or 3 or 4 or 5 or 6 or 7 or 8 or 9 or 10 or 11 or 12 or 13 or 14 or 15 or 16 or 17 or 18 or 19 or 20 141530
22 benzodiazepines/ or alprazolam/ or benzodiazepinones/ or chlordiazepoxide/ or clobazam/ or clorazepate dipotassium/ or estazolam/ or medazepam/ or midazolam/ or olanzapine/ or triazolam/ or diltiazem/ 52835
23 (benzodiazepine* or alprazolam or bromazepam or clonazepam or diazepam or flumazenil or flunitrazepam or flurazepam or lorazepam or nitrazepam or oxazepam or temazepam or chlordiazepoxide or clobazam or clorazepate or estazolam or midazolam or nayzilam or triazolam).mp. [mp=title, book title, abstract, original title, name of substance word, subject heading word, floating sub-heading word, keyword heading word, organism supplementary concept word, protocol supplementary concept word, rare disease supplementary concept word, unique identifier, synonyms, population supplementary concept word, anatomy supplementary concept word] 96861
24 Adrenergic beta-Antagonists/ 42984
25 (beta block* or acebutolol or atenolol or bisoprolol or metoprolol or nadolol or nebivolol or propranolol or sotalol or betaxolol or pindolol or alprenolol or timolol).mp. [mp=title, book title, abstract, original title, name of substance word, subject heading word, floating sub-heading word, keyword heading word, organism supplementary concept word, protocol supplementary concept word, rare disease supplementary concept word, unique identifier, synonyms, population supplementary concept word, anatomy supplementary concept word] 101011
26 calcium channel agonists/ or calcium channel blockers/ or amlodipine/ or amlodipine besylate, olmesartan medoxomil drug combination/ or amrinone/ or bencyclane/ or bepridil/ or carvedilol/ or cinnarizine/ or conotoxins/ or diltiazem/ or felodipine/ or fendiline/ or flunarizine/ or gallopamil/ or isradipine/ or lamotrigine/ or

lidoflazine/ or mibefradil/ or nicardipine/ or nifedipine/ or nimodipine/ or nisoldipine/ or nitrendipine/ or perhexiline/ or prenylamine/ or risedronic acid/ or tiapamil hydrochloride/ or verapamil/ 85760
27 (calcium channel block* or calcium channel antag* or amlodipine or diltiazem or felodipine or isradipine or nicardipine or nifedipine or verapamil).mp. [mp=title, book title, abstract, original title, name of substance word, subject heading word, floating sub-heading word, keyword heading word, organism supplementary concept word, protocol supplementary concept word, rare disease supplementary concept word, unique identifier, synonyms, population supplementary concept word, anatomy supplementary concept word] 88699
28 cocaine/ or crack cocaine/ 28191
29 cocaine.mp. 46135
30 Cyanides/ 13163
31 Nitroprusside/ae, po, to [Adverse Effects, Poisoning, Toxicity] 501
32 cobamides/ or hydroxocobalamin/ 2126
33 Nitrites/ 22519
34 (cyanide or nitroprusside or nitrite or hydroxocobalamin or cobinamide).mp. 76063
35 oleander.mp. or Nerium/ 660
36 Bufanolides/ 1501
37 (bufadeinolide or cardenolide).mp. 506
38 lily of the valley.mp. or Convallaria/ 209
39 foxglove.mp. or Digitalis/ 3422
40 Immunoglobulin Fab Fragments/ 12665
41 (digibind or digifab or digitab).mp. 120
42 anesthetics, local/ or benzocaine/ or bupivacaine/ or carticaine/ or dibucaine/ or etidocaine/ or levobupivacaine/ or lidocaine/ or lidocaine, prilocaine drug combination/ or mepivacaine/ or prilocaine/ or procaine/ or propoxycaine/ or ropivacaine/ or tetracaine/ or trimecaine/ 73210
43 (local anesthet* or benzocaine or bupivacaine or carbocaine or chloroprocaine or lidocaine or mepivacaine or procaine or prilocaine or proparacaine or ropivacaine).mp. 78034
44 Nerve Block/ae [Adverse Effects] 3416
45 Anesthesia, Dental/ae, co [Adverse Effects, Complications] 1720
46 bier* block.mp. 194
47 Fat Emulsions, Intravenous/ 4423
48 (lipid emulsion* or intralipid or lipid rescue).mp. 5847
49 Soybean Oil/tu [Therapeutic Use] 189
50 Methemoglobinemia/ 3483
51 (methemoglobin* or methaemoglobin*).mp. 9399
52 Organophosphate Poisoning/ 2375
53 Organophosphates/ae, to [Adverse Effects, Toxicity] 1560
54 organophosphorus compounds/ or organophosphates/ or carbamyl phosphate/ or chlorfenvinphos/ or dichlorvos/ or mevinphos/ or monocrotophos/ or naled/ or organothiophosphates/ or amifostine/ or chlorpyrifos/ or coumaphos/ or cystaphos/ or diazinon/ or dimethoate/ or disulfoton/ or echothiophate iodide/ or fenitrothion/ or fenthion/ or malathion/ or parathion/ or methyl parathion/ or phorate/ or

phosmet/ or temefos/ or paraoxon/ or phosphamidon/ or tritolyl phosphates/ or aminoethylphosphonic acid/ or armin/ or cidofovir/ or diphosphonates/ or organofluorophosphonates/ or isoflurophate/ or sarin/ or soman/ or organothiophosphonates/ or fonofos/ or leptophos/ or "phenylphosphonothioic acid, 2-ethyl 2-(4-nitrophenyl) ester"/ or azinphosmethyl/ 73111
55 Nerve Agents/ 566
56 (ortanophosphate* or organothiophosphate* or organophosphorous* or nerve agent* or nerve gas*).mp. 5175
57 (carbamyl* or chlorfenvinphos or dichlorvos or mevinphos or amifostine or chlorpyrifos or coumaphos or diazinon or fenitrothion or fenthion or malathion).mp. or *parathion/ or phorate.mp. or phosmet.mp. or temefos.mp. or paraxon*.mp. or phosphamidon.mp. or cidofovir.mp. or isoflurophate.mp. or sarin.mp. or soman.mp. or vx.mp. or novichok.mp. 35645
58 carbamates/ or aldicarb/ or benomyl/ or carbamyl phosphate/ or carbaryl/ 17406
59 oximes/ or obidoxime chloride/ or pralidoxime compounds/ or trimedoxime/ 10627
60 (2-pam or 2pam or protopam or pralidoxime or HI-6 or asoxime).mp. 2252
61 obidoxime.mp. 599
62 Antidepressive Agents, Tricyclic/ 10739
63 (tricyclic antidepress* or cyclic antidepress*).mp. 10163
64 amitriptyline/ or nortriptyline/ or protriptyline/ 8731
65 Amoxapine/ 343
66 clomipramine/ or desipramine/ or imipramine/ or lofepramine/ or mianserin/ or trimipramine/ 19648
67 Doxepin/ 876
68 Maprotiline/ 878
69 Sodium Channel Blockers/ 4583
70 (sodium channel block* or sodium channel antag*).mp. 7320
71 (bicyclic antidepressant* or tetracyclic antidepressant*).mp. 339
72 (amitriptyline or amoxapine or desipramine or doxepin or imipramine or nortriptyline or protriptyline or trimipramine or maprotiline or carbamazepine or chloroquine or diphenhydramine or flecainide or hydroxychloroquine or lamotrigine or lacosamide or propafenone or quinine or quinidine or thioridazine or taxus or yew or topiramate or venlafaxine or zonisamide or bupropion).mp. 132585
73 Selective Serotonin Reuptake Inhibitors/ 22411
74 "Serotonin and Noradrenaline Reuptake Inhibitors"/ or Antidepressive Agents, Second-Generation/ or Antidepressive Agents/ 61042
75 (SSRI or SNRI or (Serotonin adj3 inhib*)).mp. [mp=title, book title, abstract, original title, name of substance word, subject heading word, floating sub-heading word, keyword heading word, organism supplementary concept word, protocol supplementary concept word, rare disease supplementary concept word, unique identifier, synonyms, population supplementary concept word, anatomy supplementary concept word] 33648
76 (citalopram or escitalopram).mp. [mp=title, book title, abstract, original title, name of substance word, subject heading word, floating sub-heading word, keyword heading word, organism supplementary concept word, protocol supplementary

concept word, rare disease supplementary concept word, unique identifier, synonyms, population supplementary concept word, anatomy supplementary concept word] 8610
77 loperamide.mp. 2339
78 lopermaide/ or citalopram/ or escitalopram/ 5836
79 sympathomimetics/ or amphetamine/ or benzphetamine/ or methamphetamine/ or methoxamine/ 35542
80 amphetamines/ or amphetamine/ or dextroamphetamine/ or 2,5-dimethoxy-4-methylamphetamine/ or p-hydroxyamphetamine/ or iofetamine/ or methamphetamine/ or 3,4-methylenedioxyamphetamine/ or n-methyl-3,4-methylenedioxyamphetamine/ or dimethoxyphenylethylamine/ or methoxamine/ or p-methoxy-n-methylphenethylamine/ 45076
81 excitatory amino acid agents/ or excitatory amino acid agonists/ 8440
82 Central Nervous System Stimulants/ 23538
83 *amphetamine/ or dexedrine.mp. or procentra.mp. or *cathinone/ or *methylpropion/ or methylone.mp. or pyrovalerone.mp. or methylphenidate.mp. or daytrana.mp. or concerta.mp. or jurnay.mp. or methylin.mp. or ritalin.mp. 17344
84 p-chloroamphetamine/ or 2,5-dimethoxy-4-methylamphetamine/ or 3,4-methylenedioxyamphetamine/ or n-methyl-3,4-methylenedioxyamphetamine/ 5827
85 exp Substance-Related Disorders/co [complications] 56044
86 exp Inhalant Abuse/co 82
87 exp Hydrocarbons, Halogenated/po 3144
88 Fluorocarbons/ae, po, to 4206
89 ((Volatile substance* or Inhalant* or Hydrocarbon* or flurocarbon*) adj2 ("use" or usag* or abus* misus* or inhal*)).ab,kf,ti. 4744
90 ((sniff* or inhal* or volatile) adj4 (Sudden adj3 death)).ab,kf,ti. 49
91 Carbon Monoxide Poisoning/ 5984
92 Carbon monoxide/ 20019
93 carbon monoxide.mp. 37518
94 hydrofluoric acid/ 1593
95 (hydrofluoric acid* or hydrogen fluorid*).mp. [mp=title, book title, abstract, original title, name of substance word, subject heading word, floating sub-heading word, keyword heading word, organism supplementary concept word, protocol supplementary concept word, rare disease supplementary concept word, unique identifier, synonyms, population supplementary concept word, anatomy supplementary concept word] 2914
96 cnidaria/ or cubozoa/ or hydrozoa/ or scyphozoa/ 5573
97 Cnidarian venoms/ 1924
98 (jellyfish or jelly-fish or irukandji).mp. [mp=title, book title, abstract, original title, name of substance word, subject heading word, floating sub-heading word, keyword heading word, organism supplementary concept word, protocol supplementary concept word, rare disease supplementary concept word, unique identifier, synonyms, population supplementary concept word, anatomy supplementary concept word] 2635
99 22 or 23 or 24 or 25 or 26 or 27 or 28 or 29 or 30 or 31 or 32 or 33 or 34 or 35 or 36 or 37 or 38 or 39 or 40 or 41 or 42 or 43 or 44 or 45 or 46 or 47 or 48 or 49 or 50 or 51 or

52 or 53 or 54 or 55 or 56 or 57 or 58 or 59 or 60 or 61 or 62 or 63 or 64 or 65 or 66 or 67 or 68 or 69 or 70 or 71 or 72 or 73 or 74 or 75 or 76 or 77 or 78 or 79 or 80 or 81 or 82 or 83 or 84 or 85 or 86 or 87 or 88 or 89 or 90 or 91 or 92 or 93 or 94 or 95 or 96 or 97 or 98 1025709
100 drug overdose/ or prescription drug overuse/ 16987
101 Poisoning/ 23394
102 Toxi*.mp. 1311986
103 Poisons/ 3467
104 poison*.mp. 166548
105 overdose*.mp. 32738
106 Antidotes/ 6755
107 antidote*.mp. 11252
108 "Drug-Related Side Effects and Adverse Reactions"/ 41627
109 intoxication.mp. 52591
110 100 or 101 or 102 or 103 or 104 or 105 or 106 or 107 or 108 or 109 1500623
111 21 and 99 and 110 2157
112 exp animals/ not humans.sh. 5477450
113 111 not 112 1746
114 limit 113 to dt=20250701-20271231 36

EMBASE search

Original search

Embase <1974 to 2025 November 25>
1 exp heart arrest/ 154228
2 heart ventricle fibrillation/ 41178
3 resuscitation/ 153023
4 heart massage/ 2630
5 cardi* arrest*.mp. 100846
6 heart arrest*.mp. 107544
7 OHCA.mp. 10334
8 IHCA.mp. 1749
9 CPR.mp. 34085
10 advanced cardiac life support.mp. 2764
11 ACLS.mp. 2959
12 basic life support.mp. 4700
13 BLS.mp. 4888
14 asystol*.mp. 8831
15 pulseless electrical activity.mp. 2803
16 pulseless ventricular tachycardia.mp. 802
17 (return of circulation or return of spontaneous circulation or ROSC).mp. 18186
18 ventricular fibrillation*.mp. 31668
19 chest compression*.mp. 9061

20 1 or 2 or 3 or 4 or 5 or 6 or 7 or 8 or 9 or 10 or 11 or 12 or 13 or 14 or 15 or 16 or 17 or 18 or 19 318194
21 benzodiazepine derivative/ or alprazolam/ or chlordiazepoxide/ or clobazam/ or clorazepate dipotassium/ or estazolam/ or medazepam/ or midazolam/ or olanzapine/ or triazolam/ or diltiazem/ 235573
22 (benzodiazepine* or alprazolam or bromazepam or clonazepam or diazepam or flumazenil or flunitrazepam or flurazepam or lorazepam or nitrazepam or oxazepam or temazepam or chlordiazepoxide or clobazam or clorazepate or estazolam or midazolam or nayzilam or triazolam).mp. 312379
23 beta adrenergic receptor blocking agent/ 173896
24 (beta block* or acebutolol or atenolol or bisoprolol or metoprolol or nadolol or nebivolol or propranolol or sotalol or betaxolol or pindolol or alprenolol or timolol).mp. 254483
25 calcium channel stimulating agent/ or calcium channel blocking agent/ or amlodipine/ or amlodipine plus olmesartan/ or amlodipine besylate/ or amrinone/ or bencyclane/ or bepridil/ or carvedilol/ or cinnarizine/ or conotoxin/ or diltiazem/ or felodipine/ or fendiline/ or flunarizine/ or gallopamil/ or isradipine/ or lamotrigine/ or lidoflazine/ or mibefradil/ or nicardipine/ or nifedipine/ or nimodipine/ or nisoldipine/ or nitrendipine/ or perhexiline/ or prenylamine/ or risedronic acid/ or tiapamil/ or verapamil/ 302306
26 (calcium channel block* or calcium channel antag* or amlodipine or diltiazem or felodipine or isradipine or nicardipine or nifedipine or verapamil).mp. 230679
27 cocaine/ 67083
28 cocaine.mp. 82237
29 cyanide/ 14475
30 nitroprusside sodium/ae, to [Adverse Drug Reaction, Drug Toxicity] 986
31 cobamamide/ or hydroxocobalamin/ 4588
32 nitrate/ 70674
33 (cyanide or nitroprusside or nitrite or hydroxocobalamin or cobinamide).mp. 129967
34 oleander.mp. or Nerium/ 1470
35 bufadienolide derivative/ 526
36 (bufadeinolide or cardenolide).mp. 1510
37 lily of the valley.mp. or Convallaria/ 245
38 foxglove.mp. or digitalis/ 12918
39 "immunoglobulin F(ab) fragment"/ 9629
40 (digibind or digifab or digitab).mp. 396
41 local anesthesia/ or benzocaine/ or bupivacaine/ or articaine/ or cinchocaine/ or etidocaine/ or levobupivacaine/ or lidocaine plus prilocaine/ or lidocaine/ or mepivacaine/ or prilocaine/ or procaine/ or propoxycaine/ or ropivacaine/ or tetracaine/ or trimecaine/ 214706
42 (local anesthet* or benzocaine or bupivacaine or carbocaine or chloroprocaine or lidocaine or mepivacaine or procaine or prilocaine or proparacaine or ropivacaine).mp. 211184
43 nerve block/ae [Adverse Drug Reaction] 488
44 dental anesthesia/ae [Adverse Drug Reaction] 937
45 bier* block.mp. 357

46 lipid emulsion/ 7302
47 (lipid emulsion* or intralipid or lipid rescue).mp. 13764
48 soybean oil/dt [Drug Therapy] 221
49 methemoglobinemia/ 5986
50 (methemoglobin* or methaemoglobin*).mp. 12884
51 Organophosphate Poisoning/ 1344
52 organophosphate/ae, to [Adverse Drug Reaction, Drug Toxicity] 2271
53 organophosphorus compound/ or organophosphate/ or carbamoyl phosphate/ or clofenvinfos/ or dichlorvos/ or mevinphos/ or monocrotophos/ or naled/ or phosphorothioic acid derivative/ or amifostine/ or chlorpyrifos/ or coumafos/ or cystafos/ or dimpylate/ or dimethoate/ or disulfoton/ or ecothiopate iodide/ or fenitrothion/ or fenthion/ or malathion/ or parathion/ or methyl parathion/ or phorate/ or phosmet/ or temefos/ or paraoxon/ or phosphamidon/ or tritolyl phosphates/ or aminoethylphosphonic acid/ or armin/ or cidofovir/ or diphosphonates/ or organofluorophosphonates/ or isoflurophate/ or sarin/ or soman/ or organothiophosphonates/ or fonofos/ or leptophos/ or "phenylphosphonothioic acid, 2-ethyl 2-(4-nitrophenyl) ester"/ or azinphosmethyl/ 115887
54 nerve gas/ 1309
55 (ortanophosphate* or organothiophosphate* or organophosphorous* or nerve agent* or nerve gas*).mp. 5974
56 (carbamyl* or chlorfenvinphos or dichlorvos or mevinphos or amifostine or chlorpyrifos or coumaphos or diazinon or fenitrothion or fenthion or malathion).mp. or *parathion/ or phorate.mp. or phosmet.mp. or temefos.mp. or paraxon*.mp. or phosphamidon.mp. or cidofovir.mp. or isoflurophate.mp. or sarin.mp. or soman.mp. or vx.mp. or novichok.mp. 55968
57 carbamic acid derivative/ or aldicarb/ or benomyl/ or carbamoyl phosphate/ or carbaril/ 14670
58 oxime/ or obidoxime/ or pralidoxime/ or trimedoxime/ 7718
59 (2-pam or 2pam or protopam or pralidoxime or HI-6 or asoxime).mp. 3739
60 obidoxime.mp. 1253
61 tricyclic antidepressant agent/ 37679
62 (tricyclic antidepress* or cyclic antidepress*).mp. 43699
63 amitriptyline/ or nortriptyline/ or protriptyline/ 53173
64 clomipramine/ or desipramine/ or imipramine/ or lofepramine/ or mianserin/ or trimipramine/ 65206
65 doxepin/ 10576
66 maprotiline/ 6488
67 sodium channel blocking agent/ 4975
68 (sodium channel block* or sodium channel antag*).mp. 8098
69 (bicyclic antidepressant* or tetracyclic antidepressant*).mp. 1341
70 (amitriptyline or amoxapine or desipramine or doxepin or imipramine or nortriptyline or protriptyline or trimipramine or maprotiline or carbamazepine or chloroquine or diphenhydramine or flecainide or hydroxychloroquine or lamotrigine or lacosamide or propafenone or quinine or quinidine or thioridazine or taxus or yew or topiramate or venlafaxine or zonisamide or bupropion).mp. 402454
71 serotonin uptake inhibitor/ 58143
72 serotonin noradrenalin reuptake inhibitor/ 9776

73 serotonin noradrenalin reuptake inhibitor/ or antidepressant agent/ 135061
74 (SSRI or SNRI or (Serotonin adj3 inhib*)).mp. 77536
75 (citalopram or escitalopram).mp. 40788
76 loperamide.mp. 11998
77 loperamide/ or citalopram/ or escitalopram/ 51431
78 adrenergic receptor stimulating agent/ or amphetamine/ or benzphetamine/ or methamphetamine/ or methoxamine/ 72188
79 dimethoxy 4 methylamphetamine/ or 3,4 methylenedioxyamphetamine/ or hydroxyamphetamine/ or iofetamine/ or methamphetamine/ or n ethyl 3,4 methylenedioxyamphetamine/ or dexamphetamine/ or amphetamine/ or homoveratrylamine/ or methoxamine/ or 4 methoxy n methylphenethylamine/ 74899
80 excitatory amino acid/ 3630
81 central stimulant agent/ 19246
82 *amphetamine/ or dexedrine.mp. or procentra.mp. or *cathinone/ or *methylpropion/ or methylone.mp. or pyrovalerone.mp. or methylphenidate.mp. or daytrana.mp. or concerta.mp. or jurnay.mp. or methylin.mp. or ritalin.mp. 43760
83 chloramphetamine/ 1110
84 exp substance abuse/co [Complication] 1533
85 exp drug dependence/co [Complication] 6060
86 exp inhalant abuse/co [Complication] 10
87 exp halogenated hydrocarbon/po [Oral Drug Administration] 1
88 exp fluorocarbon/ae, to, po [Adverse Drug Reaction, Drug Toxicity, Oral Drug Administration] 683
89 ((Volatile substance* or Inhalant* or Hydrocarbon* or flurocarbon*) adj2 ("use" or usag* or abus* misus* or inhal*)).ab,kf,ti. 7751
90 ((sniff* or inhal* or volatile) adj4 (Sudden adj3 death)).ab,kf,ti. 80
91 carbon monoxide/ 48855
92 carbon monoxide intoxication/ 6472
93 carbon monoxide.mp. 68265
94 hydrofluoric acid/ 3895
95 (hydrofluoric acid* or hydrogen fluorid*).mp. 5208
96 cnidaria/ or Cubozoa/ or Hydrozoa/ or Scyphozoa/ 4775
97 coelenterate venom/ 408
98 (jellyfish or jelly-fish or irukandji).mp. 3345
99 21 or 22 or 23 or 24 or 25 or 26 or 27 or 28 or 29 or 30 or 31 or 32 or 33 or 34 or 35 or 36 or 37 or 38 or 39 or 40 or 41 or 42 or 43 or 44 or 45 or 46 or 47 or 48 or 49 or 50 or 51 or 52 or 53 or 54 or 55 or 56 or 57 or 58 or 59 or 60 or 61 or 62 or 63 or 64 or 65 or 66 or 67 or 68 or 69 or 70 or 71 or 72 or 73 or 74 or 75 or 76 or 77 or 78 or 79 or 80 or 81 or 82 or 83 or 84 or 85 or 86 or 87 or 88 or 89 or 90 or 91 or 92 or 93 or 94 or 95 or 96 or 97 or 98 2056963
100 drug overdose/ or intoxication/ 221374
101 poison/ 7711
102 toxi*.mp. 2095373
103 overdose.mp. 62462
104 poison*.mp. 148910
105 antidote/ 8279

106 antidote*.mp. 15140
107 drug toxicity/ 113850
108 intoxication.mp. 257079
109 drug intoxication/ 24108
110 100 or 101 or 102 or 103 or 104 or 105 or 106 or 107 or 108 or 109 2295129
111 20 and 99 and 110 7399
112 (rat or rats or mouse or mice or swine or porcine or murine or sheep or lambs or pigs or piglets or rabbit or rabbits or cat or cats or dog or dogs or cattle or bovine or monkey or monkeys or trout or marmoset$1).ti. and animal experiment/ 1327220
113 animal experiment/ not (human experiment/ or human/) 2806987
114 111 not (112 or 113) 6966

EMBASE search update

Embase <1974 to 2026 July 17>

1 exp heart arrest/ 162169
2 heart ventricle fibrillation/ 42330
3 resuscitation/ 159099
4 heart massage/ 2674
5 cardi* arrest*.mp. 105891
6 heart arrest*.mp. 112725
7 OHCA.mp. 11030
8 IHCA.mp. 1882
9 CPR.mp. 35656
10 advanced cardiac life support.mp. 2944
11 ACLS.mp. 3123
12 basic life support.mp. 4947
13 BLS.mp. 5152
14 asystol*.mp. 9130
15 pulseless electrical activity.mp. 2954
16 pulseless ventricular tachycardia.mp. 841
17 (return of circulation or return of spontaneous circulation or ROSC).mp. 19332
18 ventricular fibrillation*.mp. 32467
19 chest compression*.mp. 9421
20 1 or 2 or 3 or 4 or 5 or 6 or 7 or 8 or 9 or 10 or 11 or 12 or 13 or 14 or 15 or 16 or 17 or 18 or 19 331476
21 benzodiazepine derivative/ or alprazolam/ or chlordiazepoxide/ or clobazam/ or clorazepate dipotassium/ or estazolam/ or medazepam/ or midazolam/ or olanzapine/ or triazolam/ or diltiazem/ 244052
22 (benzodiazepine* or alprazolam or bromazepam or clonazepam or diazepam or flumazenil or flunitrazepam or flurazepam or lorazepam or nitrazepam or oxazepam or temazepam or chlordiazepoxide or clobazam or clorazepate or estazolam or midazolam or nayzilam or triazolam).mp. 322916
23 beta adrenergic receptor blocking agent/ 180134

24 (beta block* or acebutolol or atenolol or bisoprolol or metoprolol or nadolol or nebivolol or propranolol or sotalol or betaxolol or pindolol or alprenolol or timolol).mp. 260201

25 calcium channel stimulating agent/ or calcium channel blocking agent/ or amlodipine/ or amlodipine plus olmesartan/ or amlodipine besylate/ or amrinone/ or bencyclane/ or bepridil/ or carvedilol/ or cinnarizine/ or conotoxin/ or diltiazem/ or felodipine/ or fendiline/ or flunarizine/ or gallopamil/ or isradipine/ or lamotrigine/ or lidoflazine/ or mibefradil/ or nicardipine/ or nifedipine/ or nimodipine/ or nisoldipine/ or nitrendipine/ or perhexiline/ or prenylamine/ or risedronic acid/ or tiapamil/ or verapamil/ 310183

26 (calcium channel block* or calcium channel antag* or amlodipine or diltiazem or felodipine or isradipine or nicardipine or nifedipine or verapamil).mp. 236545

27 cocaine/ 68413

28 cocaine.mp. 83849

29 cyanide/ 14415

30 nitroprusside sodium/ae, to [Adverse Drug Reaction, Drug Toxicity] 993

31 cobamamide/ or hydroxocobalamin/ 4749

32 nitrate/ 73251

33 (cyanide or nitroprusside or nitrite or hydroxocobalamin or cobinamide).mp. 132571

34 oleander.mp. or Nerium/ 1520

35 bufadienolide derivative/ 531

36 (bufadeinolide or cardenolide).mp. 1541

37 lily of the valley.mp. or Convallaria/ 249

38 foxglove.mp. or digitalis/ 12952

39 "immunoglobulin F(ab) fragment"/ 9743

40 (digibind or digifab or digitab).mp. 409

41 local anesthesia/ or benzocaine/ or bupivacaine/ or articaine/ or cinchocaine/ or etidocaine/ or levobupivacaine/ or lidocaine plus prilocaine/ or lidocaine/ or mepivacaine/ or prilocaine/ or procaine/ or propoxycaine/ or ropivacaine/ or tetracaine/ or trimecaine/ 224158

42 (local anesthet* or benzocaine or bupivacaine or carbocaine or chloroprocaine or lidocaine or mepivacaine or procaine or prilocaine or proparacaine or ropivacaine).mp. 219471

43 nerve block/ae [Adverse Drug Reaction] 488

44 dental anesthesia/ae [Adverse Drug Reaction] 933

45 bier* block.mp. 365

46 lipid emulsion/ 7489

47 (lipid emulsion* or intralipid or lipid rescue).mp. 14071

48 soybean oil/dt [Drug Therapy] 226

49 methemoglobinemia/ 6198

50 (methemoglobin* or methaemoglobin*).mp. 13201

51 Organophosphate Poisoning/ 1424

52 organophosphate/ae, to [Adverse Drug Reaction, Drug Toxicity] 2335

53 organophosphorus compound/ or organophosphate/ or carbamoyl phosphate/ or clofenvinfos/ or dichlorvos/ or mevinphos/ or monocrotophos/ or naled/ or phosphorothioic acid derivative/ or amifostine/ or chlorpyrifos/ or coumafos/ or

cystafos/ or dimpylate/ or dimethoate/ or disulfoton/ or ecothiopate iodide/ or fenitrothion/ or fenthion/ or malathion/ or parathion/ or methyl parathion/ or phorate/ or phosmet/ or temefos/ or paraoxon/ or phosphamidon/ or tritolyl phosphates/ or aminoethylphosphonic acid/ or armin/ or cidofovir/ or diphosphonates/ or organofluorophosphonates/ or isoflurophate/ or sarin/ or soman/ or organothiophosphonates/ or fonofos/ or leptophos/ or "phenylphosphonothioic acid, 2-ethyl 2-(4-nitrophenyl) ester"/ or azinphosmethyl/ 118929
54 nerve gas/ 1379
55 (ortanophosphate* or organothiophosphate* or organophosphorous* or nerve agent* or nerve gas*).mp. 6091
56 (carbamyl* or chlorfenvinphos or dichlorvos or mevinphos or amifostine or chlorpyrifos or coumaphos or diazinon or fenitrothion or fenthion or malathion).mp. or *parathion/ or phorate.mp. or phosmet.mp. or temefos.mp. or paraxon*.mp. or phosphamidon.mp. or cidofovir.mp. or isoflurophate.mp. or sarin.mp. or soman.mp. or vx.mp. or novichok.mp. 57309
57 carbamic acid derivative/ or aldicarb/ or benomyl/ or carbamoyl phosphate/ or carbaril/ 14777
58 oxime/ or obidoxime/ or pralidoxime/ or trimedoxime/ 7921
59 (2-pam or 2pam or protopam or pralidoxime or HI-6 or asoxime).mp. 3791
60 obidoxime.mp. 1266
61 tricyclic antidepressant agent/ 38266
62 (tricyclic antidepress* or cyclic antidepress*).mp. 44343
63 amitriptyline/ or nortriptyline/ or protriptyline/ 54162
64 clomipramine/ or desipramine/ or imipramine/ or lofepramine/ or mianserin/ or trimipramine/ 65719
65 doxepin/ 10729
66 maprotiline/ 6521
67 sodium channel blocking agent/ 5155
68 (sodium channel block* or sodium channel antag*).mp. 8398
69 (bicyclic antidepressant* or tetracyclic antidepressant*).mp. 1369
70 (amitriptyline or amoxapine or desipramine or doxepin or imipramine or nortriptyline or protriptyline or trimipramine or maprotiline or carbamazepine or chloroquine or diphenhydramine or flecainide or hydroxychloroquine or lamotrigine or lacosamide or propafenone or quinine or quinidine or thioridazine or taxus or yew or topiramate or venlafaxine or zonisamide or bupropion).mp. 412852
71 serotonin uptake inhibitor/ 59808
72 serotonin noradrenalin reuptake inhibitor/ 10445
73 serotonin noradrenalin reuptake inhibitor/ or antidepressant agent/ 140484
74 (SSRI or SNRI or (Serotonin adj3 inhib*)).mp. 79915
75 (citalopram or escitalopram).mp. 42085
76 loperamide.mp. 12388
77 loperamide/ or citalopram/ or escitalopram/ 53067
78 adrenergic receptor stimulating agent/ or amphetamine/ or benzphetamine/ or methamphetamine/ or methoxamine/ 73650
79 dimethoxy 4 methylamphetamine/ or 3,4 methylenedioxyamphetamine/ or hydroxyamphetamine/ or iofetamine/ or methamphetamine/ or n ethyl 3,4 methylenedioxyamphetamine/ or dexamphetamine/ or amphetamine/ or

homoveratrylamine/ or methoxamine/ or 4 methoxy n methylphenethylamine/ 76527
80 excitatory amino acid/ 3652
81 central stimulant agent/ 19921
82 *amphetamine/ or dexedrine.mp. or procentra.mp. or *cathinone/ or *methylpropion/ or methylone.mp. or pyrovalerone.mp. or methylphenidate.mp. or daytrana.mp. or concerta.mp. or jurnay.mp. or methylin.mp. or ritalin.mp. 44522
83 chloramphetamine/ 1114
84 exp substance abuse/co [Complication] 1533
85 exp drug dependence/co [Complication] 6059
86 exp inhalant abuse/co [Complication] 10
87 exp halogenated hydrocarbon/po [Oral Drug Administration] 1
88 exp fluorocarbon/ae, to, po [Adverse Drug Reaction, Drug Toxicity, Oral Drug Administration] 727
89 ((Volatile substance* or Inhalant* or Hydrocarbon* or flurocarbon*) adj2 ("use" or usag* or abus* misus* or inhal*)).ab,kf,ti. 7966
90 ((sniff* or inhal* or volatile) adj4 (Sudden adj3 death)).ab,kf,ti. 82
91 carbon monoxide/ 50067
92 carbon monoxide intoxication/ 6667
93 carbon monoxide.mp. 70818
94 hydrofluoric acid/ 4054
95 (hydrofluoric acid* or hydrogen fluorid*).mp. 5389
96 cnidaria/ or Cubozoa/ or Hydrozoa/ or Scyphozoa/ 4960
97 coelenterate venom/ 421
98 (jellyfish or jelly-fish or irukandji).mp. 3518
99 21 or 22 or 23 or 24 or 25 or 26 or 27 or 28 or 29 or 30 or 31 or 32 or 33 or 34 or 35 or 36 or 37 or 38 or 39 or 40 or 41 or 42 or 43 or 44 or 45 or 46 or 47 or 48 or 49 or 50 or 51 or 52 or 53 or 54 or 55 or 56 or 57 or 58 or 59 or 60 or 61 or 62 or 63 or 64 or 65 or 66 or 67 or 68 or 69 or 70 or 71 or 72 or 73 or 74 or 75 or 76 or 77 or 78 or 79 or 80 or 81 or 82 or 83 or 84 or 85 or 86 or 87 or 88 or 89 or 90 or 91 or 92 or 93 or 94 or 95 or 96 or 97 or 98 2118171
100 drug overdose/ or intoxication/ 223836
101 poison/ 8139
102 toxi*.mp. 2181673
103 overdose.mp. 65379
104 poison*.mp. 154696
105 antidote/ 8606
106 antidote*.mp. 15643
107 drug toxicity/ 122276
108 intoxication.mp. 260579
109 drug intoxication/ 24510
110 100 or 101 or 102 or 103 or 104 or 105 or 106 or 107 or 108 or 109 2387542
111 20 and 99 and 110 7701
112 (rat or rats or mouse or mice or swine or porcine or murine or sheep or lambs or pigs or piglets or rabbit or rabbits or cat or cats or dog or dogs or cattle or bovine or monkey or monkeys or trout or marmoset$1).ti. and animal experiment/ 1360002
113 animal experiment/ not (human experiment/ or human/) 2889794

114 111 not (112 or 113) 7262
115 limit 114 to dd=20250701-20271231 434

Cochrane library search

For the initial search, the Cochrane search was split into four segments as the search manager was unable to manage the search strategy as a single block due to its size

Summary of results from the four search blocks- original search

	Cochrane review	Cochrane protocol	Trials	Editorial	Clinical answer	**Total**
Search block one	194	11	50	1	1	**257**
Search block two	140	9	60	0	3	**212**
Search block three	167	11	55	1	0	**234**
Search block four	0	0	0	0	0	**0**
**Total**	**501**	**31**	**165**	**2**	**4**	**703**

Total hits from initial Cochrane search- 703. Editorials and clinical answers not taken forward to screening- 697 proceeded to screening.

For the updated Cochrane search, we initially searched only for the population terms (lines 1-6 in search block one) and general terms for poisoning/ antidote/ intoxication (lines 73-83 in search block two). Given the low number of hits, we decided to screen all hits, rather than repeat all four searches.

Updated Cochrane search identified 85 hits (37 Cochrane reviews; 9 Cochrane protocols; 39 Trials).

Cochrane search block one (original search)

ID Search Hits
#1 MeSH descriptor: [Heart Arrest] explode all trees 3186
#2 MeSH descriptor: [Ventricular Fibrillation] this term only 705
#3 MeSH descriptor: [Resuscitation] this term only 995
#4 MeSH descriptor: [Heart Massage] this term only 191
#5 MeSH descriptor: [Cardiopulmonary Resuscitation] explode all trees 1841
#6 cardi* arrest* OR heart arrest* OR OHCA OR IHCA OR CPR OR advanced cardiac life support OR ACLS OR basic life support OR BLS OR asystol* OR pulseless electrical activity OR pulseless ventricular tachycardia OR return of circulation OR return of

spontaneous circulation OR ROSC OR ventricular fibrillation* or chest compression* 18780
#7 {OR #1-#6} 20388
#8 MeSH descriptor: [Benzodiazepines] this term only 2588
#9 MeSH descriptor: [Alprazolam] this term only 630
#10 MeSH descriptor: [Benzodiazepinones] this term only 359
#11 MeSH descriptor: [Chlordiazepoxide] this term only 323
#12 MeSH descriptor: [Clobazam] this term only 101
#13 MeSH descriptor: [Clorazepate Dipotassium] this term only 87
#14 MeSH descriptor: [Estazolam] this term only 50
#15 MeSH descriptor: [Medazepam] this term only 10
#16 MeSH descriptor: [Midazolam] this term only 3736
#17 MeSH descriptor: [Olanzapine] this term only 1626
#18 MeSH descriptor: [Triazolam] this term only 413
#19 MeSH descriptor: [Diltiazem] this term only 1087
#20 (benzodiazepine* or alprazolam or bromazepam or clonazepam or diazepam or flumazenil or flunitrazepam or flurazepam or lorazepam or nitrazepam or oxazepam or temazepam or chlordiazepoxide or clobazam or clorazepate or estazolam or midazolam or nayzilam or triazolam) 25851
#21 MeSH descriptor: [Adrenergic beta-1 Receptor Antagonists] this term only 265
#22 beta block* or acebutolol or atenolol or bisoprolol or metoprolol or nadolol or nebivolol or propranolol or sotalol or betaxolol or pindolol or alprenolol or timolol 25435
#23 MeSH descriptor: [Calcium Channel Agonists] this term only 52
#24 MeSH descriptor: [Calcium Channel Blockers] this term only 3457
#25 MeSH descriptor: [Amlodipine] this term only 1937
#26 MeSH descriptor: [Amlodipine Besylate, Olmesartan Medoxomil Drug Combination] this term only6
#27 MeSH descriptor: [Amrinone] this term only 65
#28 MeSH descriptor: [Bencyclane] this term only 23
#29 MeSH descriptor: [Bepridil] this term only 59
#30 MeSH descriptor: [Carvedilol] this term only 837
#31 MeSH descriptor: [Cinnarizine] this term only 126
#32 MeSH descriptor: [Conotoxins] this term only 0
#33 MeSH descriptor: [Diltiazem] this term only 1087
#34 MeSH descriptor: [Felodipine] this term only 427
#35 MeSH descriptor: [Fendiline] this term only 6
#36 MeSH descriptor: [Flunarizine] this term only 181
#37 MeSH descriptor: [Gallopamil] this term only 86
#38 MeSH descriptor: [Isradipine] this term only 315
#39 MeSH descriptor: [Lamotrigine] this term only 536
#40 MeSH descriptor: [Lidoflazine] this term only 30
#41 MeSH descriptor: [Mibefradil] this term only 70
#42 MeSH descriptor: [Nicardipine] this term only 426
#43 MeSH descriptor: [Nifedipine] this term only 2470
#44 MeSH descriptor: [Nimodipine] this term only 315
#45 MeSH descriptor: [Nisoldipine] this term only 175

#46 MeSH descriptor: [Nitrendipine] this term only 385
#47 MeSH descriptor: [Perhexiline] this term only 65
#48 MeSH descriptor: [Prenylamine] this term only 30
#49 MeSH descriptor: [Risedronic Acid] this term only 418
#50 MeSH descriptor: [Tiapamil Hydrochloride] this term only 72
#51 MeSH descriptor: [Verapamil] this term only 1402
#52 calcium channel block* or calcium channel antag* or amlodipine or diltiazem or felodipine or isradipine or nicardipine or nifedipine or verapamil 16898
#53 MeSH descriptor: [Cocaine] this term only 1048
#54 MeSH descriptor: [Crack Cocaine] this term only 92
#55 cocaine 3953
#56 MeSH descriptor: [Cyanides] this term only 26
#57 MeSH descriptor: [Nitroprusside] this term only 672
#58 MeSH descriptor: [Cobamides] this term only 7
#59 MeSH descriptor: [Hydroxocobalamin] this term only 162
#60 MeSH descriptor: [Nitrates] this term only 1061
#61 cyanide or nitroprusside or nitrite or hydroxocobalamin or cobinamide 3239
#62 oleander 18
#63 MeSH descriptor: [Nerium] this term only 0
#64 MeSH descriptor: [Bufanolides] this term only 19
#65 bufadeinolide or cardenolide 3
#66 lily of the valley 0
#67 MeSH descriptor: [Convallaria] this term only 0
#68 foxglove 7
#69 MeSH descriptor: [Digitalis] this term only 28
#70 MeSH descriptor: [Immunoglobulin Fab Fragments] this term only 723
#71 digibind or digifab or digitab 10
#72 {OR #8-#71} 74403
#73 MeSH descriptor: [Drug Overdose] this term only 350
#74 MeSH descriptor: [Prescription Drug Overuse] this term only 38
#75 MeSH descriptor: [Poisoning] this term only 272
#76 toxi* 81513
#77 MeSH descriptor: [Poisons] this term only 8
#78 poison* 3418
#79 overdose* 2596
#80 MeSH descriptor: [Antidotes] this term only 210
#81 antidote* 520
#82 MeSH descriptor: [Drug-Related Side Effects and Adverse Reactions] this term only 2336
#83 intoxication 4135
#84 {OR #73-#83} 90757
#85 #7 AND #72 AND #84 257

Cochrane search block two (original search)

ID Search Hits
#1 MeSH descriptor: [Heart Arrest] explode all trees 3186
#2 MeSH descriptor: [Ventricular Fibrillation] this term only 705
#3 MeSH descriptor: [Resuscitation] this term only 995
#4 MeSH descriptor: [Heart Massage] this term only 191
#5 MeSH descriptor: [Cardiopulmonary Resuscitation] explode all trees 1841
#6 cardi* arrest* OR heart arrest* OR OHCA OR IHCA OR CPR OR advanced cardiac life support OR ACLS OR basic life support OR BLS OR asystol* OR pulseless electrical activity OR pulseless ventricular tachycardia OR return of circulation OR return of spontaneous circulation OR ROSC OR ventricular fibrillation* or chest compression* 18780
#7 {OR #1-#6} 20388
#8 MeSH descriptor: [Anesthetics, Local] this term only 11047
#9 MeSH descriptor: [Benzocaine] this term only 248
#10 MeSH descriptor: [Bupivacaine] this term only 6955
#11 MeSH descriptor: [Carticaine] this term only 362
#12 MeSH descriptor: [Dibucaine] this term only 24
#13 MeSH descriptor: [Etidocaine] this term only 73
#14 MeSH descriptor: [Levobupivacaine] this term only 759
#15 MeSH descriptor: [Lidocaine] this term only 7554
#16 MeSH descriptor: [Lidocaine, Prilocaine Drug Combination] this term only 502
#17 MeSH descriptor: [Mepivacaine] this term only 534
#18 MeSH descriptor: [Prilocaine] this term only 884
#19 MeSH descriptor: [Procaine] this term only 312
#20 MeSH descriptor: [Propoxycaine] this term only 72
#21 MeSH descriptor: [Ropivacaine] this term only 3284
#22 MeSH descriptor: [Tetracaine] this term only 423
#23 MeSH descriptor: [Trimecaine] this term only 6
#24 local anesthet* or benzocaine or bupivacaine or carbocaine or chloroprocaine or lidocaine or mepivacaine or procaine or prilocaine or proparacaine or ropivacaine 52083
#25 MeSH descriptor: [Nerve Block] this term only 5521
#26 MeSH descriptor: [Anesthesia, Dental] this term only 1376
#27 bier* block 188
#28 MeSH descriptor: [Fat Emulsions, Intravenous] this term only 571
#29 lipid emulsion* or intralipid or lipid rescue 1832
#30 MeSH descriptor: [Soybean Oil] this term only 264
#31 MeSH descriptor: [Methemoglobinemia] this term only 36
#32 methemoglobin* or methaemoglobin* 390
#33 MeSH descriptor: [Organophosphates] this term only 168
#34 MeSH descriptor: [Organophosphate Poisoning] this term only 67
#35 MeSH descriptor: [Organophosphorus Compounds] this term only 330
#36 MeSH descriptor: [Carbamyl Phosphate] this term only 1
#37 MeSH descriptor: [Chlorfenvinphos] this term only 4
#38 MeSH descriptor: [Dichlorvos] this term only 0
#39 MeSH descriptor: [Mevinphos] this term only 0

#40 MeSH descriptor: [Monocrotophos] this term only 0
#41 MeSH descriptor: [Naled] this term only 0
#42 MeSH descriptor: [Organothiophosphates] this term only 1
#43 MeSH descriptor: [Amifostine] this term only 157
#44 MeSH descriptor: [Chlorpyrifos] this term only 10
#45 MeSH descriptor: [Coumaphos] this term only 0
#46 MeSH descriptor: [Cystaphos] this term only 1
#47 MeSH descriptor: [Diazinon] this term only 2
#48 MeSH descriptor: [Dimethoate] this term only 4
#49 MeSH descriptor: [Disulfoton] this term only 1
#50 MeSH descriptor: [Echothiophate Iodide] this term only 7
#51 MeSH descriptor: [Fenitrothion] this term only 2
#52 MeSH descriptor: [Fenthion] this term only 1
#53 MeSH descriptor: [Malathion] this term only 33
#54 MeSH descriptor: [Parathion] this term only 1
#55 MeSH descriptor: [Methyl Parathion] this term only 0
#56 MeSH descriptor: [Phorate] this term only 1
#57 MeSH descriptor: [Phosmet] this term only 0
#58 MeSH descriptor: [Temefos] this term only3
#59 MeSH descriptor: [Paraoxon] this term only 2
#60 MeSH descriptor: [Phosphamidon] this term only0
#61 MeSH descriptor: [Tritolyl Phosphates] this term only 0
#62 MeSH descriptor: [Aminoethylphosphonic Acid] this term only 0
#63 MeSH descriptor: [Armin] this term only 2
#64 MeSH descriptor: [Cidofovir] this term only 59
#65 MeSH descriptor: [Diphosphonates] this term only 1547
#66 MeSH descriptor: [Organofluorophosphonates] this term only 0
#67 MeSH descriptor: [Isoflurophate] this term only 2
#68 MeSH descriptor: [Sarin] this term only 3
#69 MeSH descriptor: [Soman] this term only 2
#70 MeSH descriptor: [Organothiophosphonates] this term only 0
#71 MeSH descriptor: [Fonofos] this term only 0
#72 MeSH descriptor: [Leptophos] this term only 0
#73 MeSH descriptor: [Phenylphosphonothioic Acid, 2-Ethyl 2-(4-Nitrophenyl) Ester] this term only 0
#74 MeSH descriptor: [Azinphosmethyl] this term only 1
#75 MeSH descriptor: [Nerve Agents] this term only 2
#76 ortanophosphate* or organothiophosphate* or organophosphorous* or nerve agent* or nerve gas* 7592
#77 carbamyl* or chlorfenvinphos or dichlorvos or mevinphos or amifostine or chlorpyrifos or coumaphos or diazinon or fenitrothion or fenthion or malathion 599
#78 *parathion or phorate or phosmet or temefos or paraxon* or phosphamidon or cidofovir or isoflurophate or sarin or soman or vx or novichok 1301
#79 {OR #8-#78} 65936
#80 MeSH descriptor: [Drug Overdose] this term only 350
#81 MeSH descriptor: [Prescription Drug Overuse] this term only 38
#82 MeSH descriptor: [Poisoning] this term only 272

#83 toxi* 81513
#84 MeSH descriptor: [Poisons] this term only 8
#85 poison* 3418
#86 overdose* 2596
#87 MeSH descriptor: [Antidotes] this term only 210
#88 antidote* 520
#89 MeSH descriptor: [Drug-Related Side Effects and Adverse Reactions] this term only 2336
#90 intoxication 4135
#91 {OR #80-#90} 90757
#92 #7 AND #79 AND #91 212

Cochrane search block three (original search)

ID Search Hits
#1 MeSH descriptor: [Heart Arrest] explode all trees 3186
#2 MeSH descriptor: [Ventricular Fibrillation] this term only 705
#3 MeSH descriptor: [Resuscitation] this term only 995
#4 MeSH descriptor: [Heart Massage] this term only 191
#5 MeSH descriptor: [Cardiopulmonary Resuscitation] explode all trees 1841
#6 cardi* arrest* OR heart arrest* OR OHCA OR IHCA OR CPR OR advanced cardiac life support OR ACLS OR basic life support OR BLS OR asystol* OR pulseless electrical activity OR pulseless ventricular tachycardia OR return of circulation OR return of spontaneous circulation OR ROSC OR ventricular fibrillation* or chest compression* 18780
#7 {OR #1-#6} 20388
#8 MeSH descriptor: [Carbamates] this term only 822
#9 MeSH descriptor: [Aldicarb] this term only0
#10 MeSH descriptor: [Benomyl] this term only 1
#11 MeSH descriptor: [Carbamyl Phosphate] this term only 1
#12 MeSH descriptor: [Carbaryl] this term only 6
#13 MeSH descriptor: [Oximes] this term only 342
#14 MeSH descriptor: [Obidoxime Chloride] this term only 3
#15 MeSH descriptor: [Pralidoxime Compounds] this term only 33
#16 MeSH descriptor: [Trimedoxime] this term only 1
#17 2*pam or 2pam or protopam or pralidoxime or HI*6 or asoxime 158
#18 obidoxime 9
#19 MeSH descriptor: [Antidepressive Agents, Tricyclic] this term only 1152
#20 tricyclic antidepress* or cyclic antidepress* 2646
#21 MeSH descriptor: [Amitriptyline] this term only 1382
#22 MeSH descriptor: [Nortriptyline] this term only 512
#23 MeSH descriptor: [Protriptyline] this term only 16
#24 MeSH descriptor: [Amoxapine] this term only 39
#25 MeSH descriptor: [Clomipramine] this term only 474
#26 MeSH descriptor: [Desipramine] this term only 464
#27 MeSH descriptor: [Imipramine] this term only 1177
#28 MeSH descriptor: [Lofepramine] this term only 30

#29 MeSH descriptor: [Mianserin] this term only 502
#30 MeSH descriptor: [Trimipramine] this term only 69
#31 MeSH descriptor: [Doxepin] this term only 195
#32 MeSH descriptor: [Maprotiline] this term only 179
#33 MeSH descriptor: [Sodium Channel Blockers] this term only 96
#34 sodium channel block* or sodium channel antag* 1009
#35 bicyclic antidepressant* or tetracyclic antidepressant* 136
#36 amitriptyline or amoxapine or desipramine or doxepin or imipramine or nortriptyline or protriptyline or trimipramine or maprotiline or carbamazepine or chloroquine or diphenhydramine or flecainide or hydroxychloroquine or lamotrigine or lacosamide or propafenone or quinine or quinidine or thioridazine or taxus or yew or topiramate or venlafaxine or zonisamide or bupropion 23691
#37 MeSH descriptor: [Selective Serotonin Reuptake Inhibitors] this term only 3522
#38 MeSH descriptor: [Serotonin and Noradrenaline Reuptake Inhibitors] this term only 88
#39 MeSH descriptor: [Antidepressive Agents] this term only 5354
#40 MeSH descriptor: [Antidepressive Agents, Second-Generation] this term only 1567
#41 SSRI or SNRI 2832
#42 Serotonin NEAR/3 inhib* 6685
#43 citalopram or escitalopram 4189
#44 loperamide 677
#45 MeSH descriptor: [Citalopram] this term only 1778
#46 MeSH descriptor: [Escitalopram] this term only 165
#47 MeSH descriptor: [Loperamide] this term only 236
#48 MeSH descriptor: [Sympathomimetics] this term only 338
#49 MeSH descriptor: [Methamphetamine] this term only 781
#50 MeSH descriptor: [Sympathomimetics] this term only 338
#51 MeSH descriptor: [Benzphetamine] this term only 3
#52 MeSH descriptor: [Methoxamine] this term only 65
#53 MeSH descriptor: [Dextroamphetamine] this term only 750
#54 MeSH descriptor: [DOM 2,5-Dimethoxy-4-Methylamphetamine] this term only 2
#55 MeSH descriptor: [p-Hydroxyamphetamine] this term only 11
#56 MeSH descriptor: [Iofetamine] this term only 19
#57 MeSH descriptor: [3,4-Methylenedioxyamphetamine] this term only 30
#58 MeSH descriptor: [N-Methyl-3,4-methylenedioxyamphetamine] this term only 284
#59 MeSH descriptor: [Dimethoxyphenylethylamine] this term only 1
#60 MeSH descriptor: [Methoxamine] this term only 65
#61 MeSH descriptor: [p-Methoxy-N-methylphenethylamine] this term only 37
#62 MeSH descriptor: [Excitatory Amino Acid Agents] this term only 7
#63 MeSH descriptor: [Excitatory Amino Acid Agonists] this term only 36
#64 MeSH descriptor: [Central Nervous System Stimulants] this term only 2851

#65 *amphetamine or dexedrine or procentra or *cathinone or *methylpropion or methylone or pyrovalerone or methylphenidate or daytrana or concerta or jurnay or methylin or ritalin 7169
#66 MeSH descriptor: [p-Chloroamphetamine] this term only 0
#67 MeSH descriptor: [DOM 2,5-Dimethoxy-4-Methylamphetamine] this term only 2
#68 MeSH descriptor: [3,4-Methylenedioxyamphetamine] this term only 30
#69 MeSH descriptor: [N-Methyl-3,4-methylenedioxyamphetamine] this term only 284
#70 MeSH descriptor: [Substance-Related Disorders] explode all trees 21875
#71 MeSH descriptor: [Inhalant Abuse] explode all trees 7
#72 MeSH descriptor: [Hydrocarbons, Halogenated] explode all trees 14952
#73 MeSH descriptor: [Fluorocarbons] this term only 234
#74 (Volatile substance* or Inhalant* or Hydrocarbon* or flurocarbon*) NEAR/2 ("use" or usag* or abus* misus* or inhal*) 14842
#75 (sniff* or inhal* or volatile) NEAR/4 (Sudden NEAR/3 death) 2
#76 MeSH descriptor: [Carbon Monoxide] this term only 675
#77 MeSH descriptor: [Carbon Monoxide Poisoning] this term only 89
#78 carbon monoxide 3407
#79 {OR #8-#78} 92404
#80 MeSH descriptor: [Drug Overdose] this term only 350
#81 MeSH descriptor: [Prescription Drug Overuse] this term only 38
#82 MeSH descriptor: [Poisoning] this term only 272
#83 toxi* 81513
#84 MeSH descriptor: [Poisons] this term only 8
#85 poison* 3418
#86 overdose* 2596
#87 MeSH descriptor: [Antidotes] this term only 210
#88 antidote* 520
#89 MeSH descriptor: [Drug-Related Side Effects and Adverse Reactions] this term only 2336
#90 intoxication 4135
#91 {OR #80-#90} 90757
#92 #7 AND #79 AND #91 234

Cochrane search block four (original search)

ID Search Hits
#1 MeSH descriptor: [Heart Arrest] explode all trees 3186
#2 MeSH descriptor: [Ventricular Fibrillation] this term only 705
#3 MeSH descriptor: [Resuscitation] this term only 995
#4 MeSH descriptor: [Heart Massage] this term only 191
#5 MeSH descriptor: [Cardiopulmonary Resuscitation] explode all trees 1841
#6 cardi* arrest* OR heart arrest* OR OHCA OR IHCA OR CPR OR advanced cardiac life support OR ACLS OR basic life support OR BLS OR asystol* OR pulseless electrical activity OR pulseless ventricular tachycardia OR return of circulation OR return of

spontaneous circulation OR ROSC OR ventricular fibrillation* or chest compression* 18780
#7 {OR #1-#6} 20388
#8 hydrofluoric acid* OR hydrogen fluorid* 435
#9 MeSH descriptor: [Hydrofluoric Acid] this term only 63
#10 jellyfish OR jelly-fish OR Irukandji 27
#11 MeSH descriptor: [Cnidaria] this term only 4
#12 MeSH descriptor: [Cubozoa] this term only 7
#13 MeSH descriptor: [Hydrozoa] this term only 1
#14 MeSH descriptor: [Scyphozoa] this term only 7
#15 MeSH descriptor: [Cnidarian Venoms] this term only 6
#16 {OR #8-#15} 470
#17 MeSH descriptor: [Drug Overdose] this term only 350
#18 MeSH descriptor: [Prescription Drug Overuse] this term only 38
#19 MeSH descriptor: [Poisoning] this term only 272
#20 toxi* 81513
#21 MeSH descriptor: [Poisons] this term only 8
#22 poison* 3418
#23 overdose* 2596
#24 MeSH descriptor: [Antidotes] this term only 210
#25 antidote* 520
#26 MeSH descriptor: [Drug-Related Side Effects and Adverse Reactions] this term only 2336
#27 intoxication 4135
#28 {OR #17-#27} 90757
#29 #7 AND #16 AND #28 0

Cochrane updated search

ID Search Hits
#1 MeSH descriptor: [Heart Arrest] explode all trees 3288
#2 MeSH descriptor: [Ventricular Fibrillation] this term only 711
#3 MeSH descriptor: [Resuscitation] this term only 1014
#4 MeSH descriptor: [Heart Massage] this term only 193
#5 MeSH descriptor: [Cardiopulmonary Resuscitation] explode all trees 1917
#6 cardi* arrest* OR heart arrest* OR OHCA OR IHCA OR CPR OR advanced cardiac life support OR ACLS OR basic life support OR BLS OR asystol* OR pulseless electrical activity OR pulseless ventricular tachycardia OR return of circulation OR return of spontaneous circulation OR ROSC OR ventricular fibrillation* or chest compression* 19508
#7 {OR #1-#6} 21141
#8 MeSH descriptor: [Drug Overdose] this term only 370
#9 MeSH descriptor: [Prescription Drug Overuse] this term only 38
#10 MeSH descriptor: [Poisoning] this term only 270
#11 toxi* 84520
#12 MeSH descriptor: [Poisons] this term only 8
#13 poison* 3566

#14 overdose* 2722
#15 MeSH descriptor: [Antidotes] this term only 213
#16 antidote* 531
#17 MeSH descriptor: [Drug-Related Side Effects and Adverse Reactions] this term only 2359
#18 intoxication 4171
#19 {OR #8-#18} 94064
#20 #7 AND #19 with Cochrane Library publication date Between Jul 2025 and Dec 2026 85

World Health Organization International Clinical Trials Registry Platform

Search date-14th March 2026; updated 22nd July 2026
(cardiac arrest OR cardiopulmonary resuscitation OR CPR) AND (poison* OR toxi* OR antidote*)
8 results

ClinicalTrials.gov

Search date-14th March 2026; updated 22nd July 2026
(cardiac arrest OR cardiopulmonary resuscitation OR CPR) AND (poison* OR toxi* OR antidote*)
1 result

Table S1: further information on study populations

Study		Age (years)	Sex	Bystander CPR	Shockable rhythm	Tracheal intubation	ROSC	Survival
Alqahatani 2019		Mean 37.4 (SD 12.3)	Male 71.6% (n=1106)	44.0% (n=680)	6.1% (n=93)	66.9% (n=1033)	Event survival 30.7% (n=470)	Hospital discharge: 8.8% (n=133)
Cole 2018		Reported in whole cohort only	Reported in whole cohort only	Not recorded	Not recorded	Not recorded	Not recorded	24.4% (n=10)
Fortin 2010								
	All patients (n=61)	Median 47 (IQR 32-60)	Male 50% (n=30) 1 case missing	Not recorded	4.9% (n=3)	Not recorded	50.8% (n=31))	8.2% (n=5)
	Adults (N=53)	Median 50 (IQR 40-62)	Male 48% (n=25) 1 case missing	Not recorded	5.8% (n=3)	Not recorded	54.7% (n=29)	9.4% (n=5)
	Children (n=8)	Median 5 (IQR 4-5)	Male 63% (n=5)	Not recorded	0% (n=0)	Not recorded	25% (n=2)	0% (n=0)
Masson 2012		Reported in whole cohort only	Reported in whole cohort only	Not recorded	Not recorded	Not recorded	Not recorded	ICU discharge: 30%
Key: ICU- Intensive Care Unit; IQR- Interquartile range; SD- standard deviation								

Table S2: GRADE evidence table

Certainty assessment							№ of patients		Effect		Certainty	Importance
№ of studies	Study design	Risk of bias	Inconsistency	Indirectness	Imprecision	Other considerations	[intervention]	[comparison]	Relative (95% CI)	Absolute (95% CI)		
**General toxicological cause- sodium bicarbonate- survival to hospital discharge**												
1	non-randomised studies	extremely serious[a]	not serious	not serious	not serious	none			**OR 0.16** (0.08 to 0.31)	**0 fewer per 1,000** (from 0 fewer to 0 fewer)	⨁◯◯◯ Very low[a]	CRITICAL
**General toxicological cause- E-CPR- survival to ICU discharge**												
1	non-randomised studies	extremely serious[a]	not serious	not serious	serious[b]	none	3/3 (100.0%)	0/7 (0.0%)	**OR 105.00** (1.71 to 6465.65)	**129 more per 1,000** (from 1 more to 901 more)[c]	⨁◯◯◯ Very low[a,b]	IMPORTANT
**Calcium-channel/ beta-blockers- IV fat emulsion- survival to hospital discharge**												
1	non-randomised studies	extremely serious[a]	not serious	serious[d]	very serious[e]	none	1/14 (7.1%)	9/27 (33.3%)	**OR 0.15** (0.02 to 1.37)	**264 fewer per 1,000** (from 323 fewer to 73 more)	⨁◯◯◯ Very low[a,d,e]	CRITICAL
**Calcium-channel/ beta-blockers- glucagon- survival to hospital discharge**												
1	non-randomised studies	extremely serious[a]	not serious	serious[d]	very serious[e]	none	1/9 (11.1%)	9/32 (28.1%)	**OR 0.32** (0.03 to 2.93)	**170 fewer per 1,000** (from 270 fewer to 253 more)	⨁◯◯◯ Very low[a,d,e]	CRITICAL
**Calcium-channel/ beta-blockers- methylene blue- survival to hospital discharge**												

Certainty assessment							№ of patients		Effect		Certainty	Importance
№ of studies	Study design	Risk of bias	Inconsistency	Indirectness	Imprecision	Other considerations	[intervention]	[comparison]	Relative (95% CI)	Absolute (95% CI)		
1	non-randomised studies	extremely serious[a]	not serious	serious[d]	very serious[e]	none	0/4 (0.0%)	10/37 (27.0%)	**OR 0.29** (0.01 to 5.89)	**173 fewer per 1,000** (from 267 fewer to 415 more)	⨁◯◯◯ Very low[a,d,e]	CRITICAL
**Calcium-channel/ beta-blockers- pacemaker- survival to hospital discharge**												
1	non-randomised studies	extremely serious[a]	not serious	serious[d]	very serious[e]	none	2/7 (28.6%)	8/34 (23.5%)	**OR 1.30** (0.21 to 8.03)	**50 more per 1,000** (from 175 fewer to 477 more)	⨁◯◯◯ Very low[a,d,e]	CRITICAL
**Calcium-channel/ beta-blockers- intra-aortic balloon pump- survival to hospital discharge**												
1	non-randomised studies	extremely serious[a]	not serious	serious[d]	very serious[e]	none	0/5 (0.0%)	10/36 (27.8%)	**OR 0.23** (0.01 to 4.53)	**197 fewer per 1,000** (from 274 fewer to 358 more)	⨁◯◯◯ Very low[a,d,e]	CRITICAL
**Calcium-channel/ beta-blockers- ECMO- survival to hospital discharge**												
1	non-randomised studies	extremely serious[a]	not serious	serious[d]	very serious[e]	none	0/2 (0.0%)	10/39 (25.6%)	**OR 0.56** (0.02 to 12.69)	**95 fewer per 1,000** (from 250 fewer to 558 more)	⨁◯◯◯ Very low[a,d,e]	CRITICAL
**Smoke inhalation/ cynaide- hydroxocobalamin- ROSC**												
1	non-randomised studies	extremely serious[a]	not serious	not serious	serious[b]	none	8/8 (100.0%)	21/45 (46.7%)	**OR 19.37** (1.05 to 355.75)	**478 more per 1,000** (from 12 more to 530 more)	⨁◯◯◯ Very low[a,b]	IMPORTANT

Certainty assessment							№ of patients		Effect		Certainty	Importance
№ of studies	Study design	Risk of bias	Inconsistency	Indirectness	Imprecision	Other considerations	[intervention]	[comparison]	Relative (95% CI)	Absolute (95% CI)		
**Smoke inhalation/ cynaide- hydroxocobalamin- survival to hospital discharge**												
1	non-randomised studies	extremely serious[a]	not serious	not serious	serious[b]	none	3/8 (37.5%)	2/45 (4.4%)	**OR 12.90** (1.72 to 96.73)	**331 more per 1,000** (from 30 more to 774 more)	⨁◯◯◯ Very low[a,b]	CRITICAL

CI: confidence interval; **OR:** odds ratio

Explanations

a. Single available study at critical risk risk of bias

b. Confidence interval extremely broad- covering moderate beneficial effect to very substantial beneficial effect

c. To calculate absolute effect, we used a control group event rate of 0.1%

d. Intervention timing unclear

e. Extremely broad confidence interval incorporating both substantial harm and potential benefit
